# Behavioral and Psychosocial Predictors of Treatment Response in Emergency Psychiatric Admissions for Substance Use Disorders in a Socioeconomically Vulnerable Region of Southeastern Romania: A Five-Year Retrospective Study

**DOI:** 10.3390/bs16091496

**Published:** 2026-08-26

**Authors:** Ionela-Violeta Ragea, Simona-Dana Mitincu-Caramfil, Rusu-Negraia Magdalena, Cătălin Pleșea-Condratovici, Valeriu Ardeleanu, Ramona-Oana Roșca, Laurențiu Drăguș, Lavinia-Alexandra Moroianu

**Affiliations:** 1 Faculty of Medicine and Pharmacy, Research Centre in the Medical-Pharmaceutical Field, “Dunărea de Jos” University, 800008 Galați, Romania; iv.dogaru@yahoo.com (I.-V.R.); simona.mitincu@ugal.ro (S.-D.M.-C.); ramona.rosca@ugal.ro (R.-O.R.); laurentiu.dragus@ugal.ro (L.D.); lavinia.moroianu@yahoo.com (L.-A.M.); 2 Faculty of Kinesiotherapy, “Dunărea de Jos” University, 800008 Galați, Romania; valeriu.ardeleanu@gmail.com

**Keywords:** addictive behavior, behavioral intervention, discharge outcome, emergency psychiatry, integrated treatment, motivational counseling, psychosocial determinants, real-world evidence, risk stratification, substance use disorders

## Abstract

Substance use disorders (SUDs) represent a major psychiatric and public health burden. Although integrated biopsychosocial approaches combining pharmacotherapy with psychosocial and behavioral interventions are recommended by international guidelines, real-world evidence on their effectiveness in acute emergency settings remains scarce, particularly in Eastern European healthcare contexts. This single-center, retrospective, observational study examined behavioral and psychosocial predictors of clinical response and compared an integrated therapeutic strategy (pharmacotherapy combined with psychosocial and behavioral interventions) with pharmacotherapy alone among all emergency psychiatric admissions for SUDs (ICD-10 F10–F19) at the Clinical Psychiatric Hospital “Elisabeta Doamna,” Galați, Romania, between January 2020 and December 2024 (N = 331; census sampling). The primary outcome was clinical status at discharge (improved/stationary/worsened). Patients receiving the integrated strategy showed a significantly higher improvement rate at discharge than those receiving pharmacotherapy alone (98.3% vs. 76.3%; OR = 17.88; 95% CI: 5.62–56.91; *p* < 0.001). A hospitalization window of 4–14 days was associated with optimal outcomes. In an exploratory logistic regression analysis, older age (≥51 years) and unemployment emerged as independent psychosocial predictors of suboptimal response. These findings suggest that integrating behavioral and psychosocial interventions with pharmacotherapy is associated with superior discharge outcomes in acute SUD management; given the observational, non-randomized design, these results should be regarded as hypothesis-generating rather than causal, pending confirmation in prospective multicenter studies before informing behaviorally informed triage and risk stratification in emergency psychiatry.

## 1. Introduction

### 1.1. Scientific Background

Substance use disorders (SUDs) represent a chronic, recurrent, and multifactorial pathology with substantial impact on population-level morbidity, mortality, and disability. Global Burden of Disease (GBD) analyses consistently highlight the contribution of alcohol and drug use to the global burden of illness, both through direct mechanisms, such as intoxication, withdrawal syndromes, and organ complications, and indirect pathways, including trauma, violence, and social vulnerability. Alcohol use disorder, in particular, remains a leading determinant of preventable mortality and hepatic disease, underscoring the need for effective and scalable clinical strategies across all levels of care. Concurrently, evolving drug market dynamics and shifting consumption patterns as reflected in recent international surveillance reports continue to exert sustained pressure on emergency services and psychiatric networks, driven by polysubstance use, the emergence of novel psychoactive substances, and compounding socioeconomic vulnerabilities (Degenhardt et al., 2018; *Global status report on alcohol and health 2018*, n.d.; *World drug report 2024*, n.d.; Murray, 2022; Rehm & Shield, 2019a; Vollset et al., 2024).

In the emergency psychiatric setting, SUD presentations are predominantly characterized by two acute clinical phenomena: acute intoxication and withdrawal syndromes, frequently complicated by psychomotor agitation, suicidal ideation, impaired judgment, somatic comorbidities, and treatment non-adherence. The World Health Organization (WHO) recommends that acute SUD management be centered on stabilization, immediate risk assessment, and early initiation of a structured therapeutic pathway designed to reduce relapse probability and readmission rates. Simultaneously, evidence from emergency medicine literature demonstrates that systematic screening for harmful substance use in emergency departments is both feasible and clinically relevant; however, outcomes depend critically on the integration of screening with brief interventions, treatment referral, and continuity of care (K. Hawk & D’Onofrio, 2018; Moe et al., 2024). This “therapeutic chain” logic is particularly important in emergency psychiatry, where clinical severity and case complexity demand standardized yet adaptable interventions.

### 1.2. The Role of Comorbidity and Integrated Care

A major obstacle in acute SUD management is psychiatric comorbidity, the so-called “dual diagnosis”, which significantly influences clinical presentation, pharmacotherapy tolerance, treatment trajectory, and post-discharge prognosis. Transnational data from the WHO World Mental Health surveys suggest a relevant prevalence of comorbidities alongside pronounced deficits in access to specialized treatment, with considerable variation across healthcare systems (Harris et al., 2019). In parallel, evidence on detoxification in dual-diagnosis contexts indicates that psychiatric comorbidity modulates inpatient outcomes, justifying the integration of psychiatric assessment and psychosocial interventions from the acute phase onward (A. Davis et al., 2023). Emerging evidence further suggests that psychiatric comorbidity in medically complex patients, including those with metabolic and oncological conditions, involves neuroinflammatory mechanisms that may modulate treatment response, underscoring the need for biologically informed integrated assessments in vulnerable populations (Pâslaru et al., 2025). Integrated treatment models in vulnerable populations, such as young patients with a first psychotic episode and concurrent substance use, further support the feasibility of combined therapeutic architectures and their potential to improve clinical outcomes (Herman et al., 2023).

International guidelines from the WHO, the European Monitoring Centre for Drugs and Drug Addiction (EMCDDA), and the National Institute for Health and Care Excellence (NICE) consistently emphasize the need for a multidimensional approach in acute SUD management—one that incorporates validated screening instruments (AUDIT, CAGE-AID, SDS), evidence-based pharmacotherapy, and structured psychosocial support (European Union Drugs Agency, 2024; *International standards for the treatment of drug use disorders*, n.d.; NICE, 2011; Nana Wilson et al., 2020; Rehm et al., 2017; Rehm & Shield, 2019b). This integrated framework is grounded in the recognition that SUDs are biopsychosocial in nature; pharmacological stabilization alone, while necessary, may be insufficient when behavioral, social, and healthcare access factors exert an equally determinant influence on clinical trajectory (Crepet & Sargent, 2022; K. F. Hawk et al., 2019; Jemberie et al., 2020; Mendiola et al., 2018). At the biomarker level, neurochemical indicators such as serum serotonin have been explored as potential screening tools for affective comorbidities in metabolically vulnerable populations, illustrating the expanding scope of integrated biological assessment in psychiatric practice (L.-A. Moroianu et al., 2022).

### 1.3. Evidence Gaps in Emergency Psychiatric Practice

Despite broad consensus on the theoretical merits of integrated care, the pragmatic benefit of a combined therapeutic strategy, defined as pharmacotherapy augmented by at least one non-pharmacological component and/or structured biological/paraclinical assessment, compared to pharmacotherapy alone, remains incompletely quantified in real-world emergency psychiatric settings. Several evidence gaps are particularly relevant. First, most available data derive from outpatient addiction programs or controlled research environments, limiting their direct applicability to the complex, resource-constrained context of acute psychiatric admissions (European Union Drugs Agency, 2024; Nana Wilson et al., 2020; Rehm et al., 2017; Rehm & Shield, 2019b). Second, a meaningful gap persists in the standardized operationalization of outcome indicators used to evaluate and monitor acute SUD episodes in hospital registries, an issue with direct implications for clinical audit and inter-service comparability (Rowe et al., 2017; Snow et al., 2021; Vivolo-Kantor et al., 2021). Third, although recent literature supports the feasibility of brief, risk-oriented care packages that combine immediate clinical intervention with educational and preventive components, including emergency-department naloxone distribution programs, the real-world comparative effectiveness of structured integrated strategies on discharge outcomes has not been systematically evaluated in Eastern European psychiatric settings (Barrio & Gual, 2016; Black et al., 2022; E. L. Davis et al., 2020; Farrugia et al., 2019; Kestler et al., 2017; McDonald & Strang, 2016; Park et al., 2020).

### 1.4. Study Rationale and Objectives

Romania presents a specific epidemiological context: national data from the National Anti-Drug Agency (ANA) indicate increasing substance use among younger populations, with alcohol use disorder representing the dominant pathology in emergency psychiatric admissions, particularly in socioeconomically vulnerable regions (Agenția Națională Antidrog [ANA], 2023). The “Elisabeta Doamna” Psychiatric Hospital in Galaţi operates in a resource-limited region of southeastern Romania, offering a clinically relevant and ecologically valid setting for real-world assessment of acute SUD management.

Against this background, the present single-center retrospective observational study analyzes five years of emergency psychiatric admissions (January 2020–December 2024), with a focus on the comparative effectiveness of two management strategies: pharmacotherapy alone versus an integrated therapeutic approach (pharmacotherapy combined with at least one non-pharmacological intervention and/or diagnostic/procedural component). The primary objective is to evaluate the association between therapeutic strategy and clinical outcome at discharge, operationalized through the Global Overall Severity Score (GOSS). Secondary objectives include characterizing the sociodemographic and nosological profile of the cohort, identifying predictors of suboptimal treatment response, and exploring the relationship between hospitalization duration and discharge outcome.

The central research hypothesis is that, in a real-world emergency psychiatric setting, integrated management is associated with a significantly higher probability of clinical improvement at discharge compared to pharmacotherapy alone.

## 2. Materials and Methods

### 2.1. Study Design and Institutional Setting

This study was designed as a single-center, retrospective, observational investigation aimed at characterizing and evaluating the acute management of patients hospitalized on an emergency basis for substance use disorders (SUDs). Cases were operationally defined using ICD-10 codes F10–F19 (mental and behavioral disorders due to psychoactive substance use), ensuring internationally reproducible and comparable case identification. The study period encompassed January 2020 through December 2024, yielding a total of 331 emergency admissions, of which 294 carried an F10–F19 code as the primary diagnosis and 37 as a secondary diagnosis.

The study was conducted at Psychiatric Hospital “Elisabeta Doamna”, Galaţi, Romania, a unit with an operational role in the management of acute psychiatric pathology and emergency presentations. This real-world setting was deliberately chosen because therapeutic decisions are made under emergency-specific clinical constraints, symptom severity, the need for somatic and psychological stabilization, comorbidities, available resources, and time pressure, and clinical management frequently requires an interdisciplinary approach. ICD-10 diagnostic delineation supports standardization and comparability; findings can furthermore be contextualized in conceptual continuity with ICD-11 updates concerning substance use disorders and addictive behaviors.

From an exposure perspective, two distinct acute management patterns were operationalized based on the clinical documentation reviewed: (1) pharmacotherapy-alone intervention, an approach focused on immediate symptomatic control; and (2) integrated intervention, defined as pharmacotherapy combined with at least one additional non-pharmacological component documented in the clinical records, either a psychosocial/behavioral intervention (crisis-supportive psychotherapy, motivational counseling, psychoeducation) or a comprehensive paraclinical assessment informing the therapeutic decision. It should be noted that these two types of components are conceptually distinct: consequently, patients receiving pharmacotherapy plus clinically relevant investigations, but without a documented psychosocial or behavioral intervention, were also classified in the integrated group. This compositional heterogeneity of the exposure is explicitly addressed as a limitation in Section 4.3. This dichotomy captures the distinction between symptom-targeted stabilization and complex stabilization, the latter incorporating psychosocial and supportive measures in accordance with evidence supporting the value of structured non-pharmacological interventions in SUDs (Chrostek & Panasiuk, 2014; Chua et al., 2022). By virtue of its retrospective design, the study adheres to the real-world evidence paradigm, drawing on information derived from documented clinical trajectories and previously applied medical decisions, without interfering with the therapeutic conduct.

Because this study is retrospective and observational, allocation to the pharmacotherapy-alone versus integrated strategy was not randomized. Treatment assignment reflected the on-call psychiatrist’s clinical judgment at the moment of emergency presentation, informed by the documented characteristics of the acute episode (severity of withdrawal or intoxication, level of agitation, patient cooperation, perceived need for somatic screening, and presence of psychiatric or somatic comorbidities suggesting complex stabilization), together with contextual constraints of the emergency setting (availability of psychology, social work, and rehabilitation staff at the time of admission; workload; and time pressure). In practice, pharmacotherapy alone was more often documented when immediate symptomatic control was judged sufficient, when the patient declined or could not engage with psychosocial components, or when non-pharmacological personnel were not available during the admission window. These decision drivers are captured only indirectly through routine clinical records; their potential to introduce confounding by indication is therefore explicitly acknowledged and discussed in Section 4.3.

### 2.2. Data Sources and Collection Procedure

Data were obtained by retrospective extraction from primary clinical documents routinely used in emergency psychiatric care: the General Clinical Observation File (GCOF; Romanian: Fișa de Observație Clinică Generală, F.O.C.G.) and the Emergency Registry (ER; Romanian: Registrul de Evidență a Urgențelor, REU). The GCOF served as the primary source for sociodemographic and clinical characteristics, ICD-10 diagnoses (primary/secondary), clinical and paraclinical interventions, performed procedures, and discharge evolution; the ER was used complementarily to identify and verify emergency presentations and the chronological elements of the acute episode.

Extraction followed a standardized schema, with variables operationalized and coded into a uniform numeric format to ensure internal comparability and reproducible statistical processing. The workflow comprised: (i) retrieval of variables from the GCOF/ER and initial coding in Microsoft Excel; (ii) transfer to an IBM SPSS Statistics analysis database with variable property definitions (type, level of measurement, labels, and permissible values); (iii) flagging of missing values and generation of preliminary frequency reports; (iv) logical error checking (e.g., age outside the eligibility range, invalid ICD-10 codes) and correction, or—where correction was not possible—retention as missing; and (v) iterative updating of the coding dictionary when atypical cases were encountered.

A centralized database in tabular format (Microsoft Excel) was structured across thematic sheets covering demographics, diagnoses, investigations/procedures, pharmacological treatment, comorbidities, length of stay, and treatment response. Categorical variables were numerically coded using pre-established dictionaries; continuous variables (age, length of stay, paraclinical parameters) were entered numerically with validation rules (including clinical plausibility; for age, the reference range was 14–76 years). The extracted variable set systematically encompassed: (1) sociodemographic data (age, sex, area of residence, occupational status); (2) primary and, where applicable, secondary ICD-10 diagnosis; (3) details of the emergency presentation; (4) clinical and paraclinical investigations, including laboratory results (normal/pathological categories) and imaging studies relevant to the therapeutic decision; (5) coded procedures including DRG components (Diagnosis-Related Groups) as documented; (6) initial pharmacological treatment (agent, dose, frequency/duration or number of administrations); (7) significant comorbidities; (8) admission and discharge dates for length-of-stay calculation; and (9) clinical response assessment at discharge (categories: ‘improved’, ‘stationary’, ‘worsened’, rarely ‘recovered’).

Data quality assurance was ensured through complementary measures at both entry and verification stages: an ad-hoc coding form in Microsoft Excel incorporating drop-down lists for categorical variables (to minimize typographical errors) and automated alerts for implausible values (e.g., age below the eligibility threshold or extreme lengths of stay). Preliminary frequency/descriptive statistics runs were then performed to detect inconsistencies, followed by manual re-verification against the GCOF for each identified discrepancy. Where information could not be reconstructed from primary documents, the case was excluded solely from the analysis of that variable, without being removed from the general sample if the remaining data were complete. Additionally, quality control included double-entry verification (parallel entry by two reviewers followed by reconciliation of differences) and a random sub-sample check (15% of files) to assess concordance between extracted data and original documents, with a target concordance threshold of ≥98%.

Missing values documented in the clinical records were retained as such and managed in IBM SPSS Statistics v. 26 via resolute ‘missing’ codes, to prevent their inclusion in descriptive or inferential estimates and the consequent distortion of results. In compliance with ethical and data-protection requirements, following extraction, anonymization was applied by numeric code (ID), without retention of direct identifying elements (name, Personal Identification Number, address) in the analytical dataset. The database was password-protected (access restricted; encrypted/secured storage for exported files), and the entire process was conducted with approval from the hospital ethics committee and in accordance with the General Data Protection Regulation (GDPR) and applicable national legislation.

### 2.3. Study Population, Sample, and Eligibility Criteria

The population of interest was defined as all emergency hospitalizations occurring between 1 January 2020 and 31 December 2024 at Psychiatric Hospital “Elisabeta Doamna” Galaţi, for which a diagnosis within the ICD-10 F10–F19 series was documented either as the primary or secondary diagnosis. The sampling strategy was exhaustive (census), aiming to include all eligible cases within the reference period in order to maximize ecological validity and to reduce the risk of selection bias inherent to conventional sub-sampling approaches.

The final analytical sample comprised N = 331 cases, of which 294 carried an F10–F19 code as the primary diagnosis and 37 as a secondary diagnosis. In terms of population profile, the sample exhibited a predominant male composition (n = 297; 89.7%) and a majority urban provenance (n = 189; 57.1%). The age distribution was as follows: 14–18 years (n = 18; 5.4%), 19–25 years (n = 70; 21.1%), 26–35 years (n = 89; 26.9%), 36–50 years (n = 110; 33.2%), and ≥51 years (n = 44; 13.3%). Regarding occupational status, the majority of cases were registered as unemployed (n = 260; 78.5%), followed by employed individuals (n = 28; 8.5%) and old-age pensioners (n = 28; 8.5%). Clinically, the predominant primary diagnosis was F10.2 (alcohol dependence syndrome), and comorbidities (psychiatric and/or somatic) were documented in 152 patients (45.9%).

The eligibility criteria are summarized in Table 1.

### 2.4. Study Variables and Operationalization

The variable set was constructed to capture, within a pragmatic logic, the relationships among the patient’s sociodemographic and clinical profile, the type of acute emergency management implemented, and the clinical outcome at discharge. All variables were extracted from the GCOF/ER, converted to a standardized numeric format, and parameterized within the statistical software by specifying type, level of measurement, and the domain of permissible values; missing values were systematically flagged, and internal consistency was verified through logical consistency checks (e.g., ages outside the eligible range or invalid ICD codes).

Sociodemographic variables included age (recorded numerically and used both as a continuous variable and in clinically relevant age groups), sex, area of residence, and occupational status, to the extent that these were fully documented in the primary records. These dimensions were treated as primary explanatory factors in the analyses of therapeutic response stratification, given their role in service access, psychosocial vulnerability, and adherence during the acute phase.

Clinical variables were operationalized by coding the ICD-10 diagnosis from the F10–F19 series, distinguishing its position as primary versus secondary diagnosis to maintain the clinical detail of the admission reason and the associated psychopathological context. Documented comorbidities and available clinical and paraclinical elements were also recorded as indicators of case complexity and the need for extended monitoring in an emergency setting. Comorbidities and the need for polypharmacotherapy were considered potential confounders since they may influence both the choice of therapeutic strategy and the likelihood of a suboptimal response.

Therapeutic variables were defined through an operational classification of interventions applied in the emergency setting, aligned with ICD-10-AM standards and RO.vi.DRG guidelines to ensure comparability and reproducibility. The pharmacological component encompassed the administration of specific medication for withdrawal and acute symptom control (e.g., benzodiazepines including diazepam, cinolazepam, clonazepam), substitution therapies (methadone or buprenorphine), antipsychotics, and adjuvant agents used in current practice (e.g., B-complex vitamins, silymarin, metoprolol). The non-pharmacological component included both diagnostic and monitoring procedures (laboratory analyses, paraclinical investigations such as ECG, CT, ultrasound, and MRI, with results recorded as normal or pathological) and DRG-codifiable procedures (crisis supportive psychotherapy, psychiatric assessments, motivational counselling, psychoeducation, nutritional assessments, and functional rehabilitation programs).

On this basis, the primary exposure was operationalized dichotomously, reflecting two management modalities identifiable in the clinical documents: pharmacotherapy alone versus integrated intervention, defined as pharmacotherapy combined with at least one documented non-pharmacological component—psychosocial/behavioral intervention and/or comprehensive paraclinical assessment (see Section 2.1 for the conceptual distinction between these components). The full variable set and operationalization details are summarized in Table 2.

### 2.5. Outcome Definitions and Discharge Assessment Criteria

The primary outcome indicator was the clinical status at discharge, operationalized through a Global Overall Severity Score (GOSS). It should be emphasized that GOSS, as applied here, is not a formally validated psychometric instrument but a pragmatic, study-specific classification developed for this retrospective analysis to synthesize the direction and magnitude of clinical change during the acute episode. GOSS categorization was derived from the documented discharge conclusions in the General Clinical Observation File and coded by the first author together with a second reviewer, under the supervision of the senior investigator, by comparing symptom severity and global functioning at discharge with the status recorded at admission, in the context of the implemented management strategy and the documented degree of cooperation/adherence throughout hospitalization. Pre-specified percentage thresholds (Table 3) served as operational benchmarks to standardize classification decisions in a retrospective context, without substituting for the clinical judgment documented in the observation files. Outcome coding was embedded in the study-wide double-entry quality-control procedure described in Section 2.2 (independent parallel coding by the two reviewers, reconciliation of discrepancies against source records, and re-verification of a random 15% of files against original documentation, with a target concordance threshold of ≥98%). Two constraints should be acknowledged: because the therapeutic strategy is documented within the same records used to derive the outcome, coders could not be blinded to treatment allocation; and a formal inter-rater agreement coefficient (e.g., Cohen’s κ) was not computed as part of the original design. Both constraints are addressed as limitations in Section 4.3. In this framework, GOSS was treated as an ordinal outcome with three levels, reflecting the direction and magnitude of clinical change during emergency hospitalization. The GOSS categories and their operational definitions are presented in Table 3.

For inferential analyses, this outcome allowed both between-group comparisons in an ordinal structure and clinical-interpretability-oriented recordings (e.g., distinguishing a favorable response from a suboptimal response, coded as 0 = improved vs. 1 = stationary/worsened), depending on the objectives and the operationalized hypotheses of each analysis.

The secondary outcome was length of stay, expressed in days and derived from admission and discharge dates documented in the clinical records. This variable was analyzed both as a continuous measure (for distributional and central tendency description) and through reclassification into duration categories for association testing (1–3 days, 4–7 days, 8–14 days, ≥15 days), given the frequently asymmetric distribution and the practical utility of pragmatic thresholds in interpreting resource consumption and the clinical complexity of the acute episode. Prolonged hospitalizations were interpreted as potential signals of comorbidities unidentified at admission or of withdrawal refractory to standard treatment, justifying the inclusion of length of stay both as a descriptive variable and as a mediator/marker of severity in inferential analyses.

### 2.6. Withdrawal Management and Clinical Rationale for Interventions

Although the retrospective design does not permit comparison of randomized protocols, the therapeutic strategies applied in the emergency setting were interpreted by reference to standard clinical reasoning and best practices described in the specialist literature. In emergency psychiatry, withdrawal syndromes and acute intoxications realistically impose a sequence of overlapping objectives: immediate stabilization of vital and behavioral risk, pharmacological control of dominant symptoms, monitoring of clinical evolution, and integration of supportive/non-pharmacological components that enhance safety and facilitate patient engagement in continued treatment. This logic is congruent with the practice described in the analysis of the institutional setting, where pharmacological interventions (sedatives, antipsychotics, withdrawal-specific treatments, antidotes such as naloxone in opioid overdose) are frequently complemented by short-term psychosocial measures, monitoring, and supportive care (hydration, metabolic corrections), as well as early planning of the post-acute trajectory.

Within this framework, withdrawal management is understood as a severity- and risk-stratified intervention in which pharmacological treatment serves to reduce neurovegetative hyperexcitability, agitation, anxiety, insomnia, and acute complications, while monitoring seeks early detection of clinical deterioration, somatic complications, and comorbidities that may modify the therapeutic approach. Although sometimes perceived as merely additive, the non-pharmacological component has a distinct clinical function in the emergency setting: it optimizes de-escalation, increases cooperation, reduces risk behaviors, and supports better-informed decisions (through psychoeducation, brief motivational counselling, and reorientation techniques), aspects compatible with the integrative approach to addiction as a biopsychosocial disorder.

For alcohol withdrawal, clinical guidelines and reviews highlight the importance of risk assessment, a staged therapeutic approach, and safety measures, given the potential for severe complications (e.g., seizures, delirium tremens, hydro-electrolytic imbalances, autonomic instability) and the need to adapt the intervention to the severity of presentation and the patient’s somatic vulnerabilities. In the practice reflected by the clinical documents, this reasoning justifies the combination of prompt symptomatic control with targeted clinical, and paraclinical investigations oriented towards ruling out organic causes and assessing complications (e.g., biological parameters, cardiovascular assessment), as well as supplementation of the therapeutic approach with supportive measures when the clinical picture warrants it. Similarly, in opioid pathology, the emergency may be dominated by the risk of respiratory depression or mixed presentations (polysubstance use, psychiatric comorbidities), necessitating the integration of specific interventions (opioid antagonist/antidote when indicated) with monitoring and discharge risk-reduction measures.

Table 4 below summarizes the principal pharmacological classes documented in this emergency setting, mapped to their primary clinical indications and the dominant ICD-10 diagnostic groups in the cohort. All 331 patients (100%) received pharmacological treatment; however, the retrospective clinical records documented pharmacological interventions at the level of DRG-codifiable procedures (administered vs. not administered) rather than at the level of individual drug class, dose, or duration per patient. A quantitative frequency table of specific drug classes stratified by ICD-10 diagnosis could therefore not be reconstructed, and the mapping below reflects the general clinical practice of the institution as documented in the observation files rather than patient-level prescription data.

### 2.7. Statistical Analysis and Software

Statistical processing and analysis were performed using IBM SPSS Statistics 26.0, following an initial stage of database organization and validation in Microsoft Excel. Variables were transcribed to a standardized numeric format using coding dictionaries and value labels, with consistency checks and validation rules to prevent data entry errors; the dataset was subsequently imported into SPSS with variable property definitions (type, level of measurement, permissible ranges). The analysis comprised both a descriptive and an inferential component. An overview of the statistical methods applied is provided in Table 5.

The association was quantified through effect size estimation, including the odds ratio (OR) and 95% confidence intervals. For the identification of predictors of suboptimal response, binary logistic regression models were estimated, and the discriminatory performance of the models was evaluated using ROC curve analysis and the area under the curve (AUC), together with classification indicators (sensitivity, specificity). Given the low number of suboptimal events (n = 14) relative to the number of candidate predictors, the logistic regression was pre-specified as an exploratory, hypothesis-generating analysis rather than a confirmatory one. The events-per-variable ratio (~3.5) is below the conventional threshold of ten; consequently, coefficient estimates should be interpreted with caution, and the model is reported primarily to identify candidate risk markers for prospective validation rather than to establish definitive effect magnitudes. The significance threshold was set at α = 0.05, two-tailed.

A formal strategy-adjusted model (including treatment strategy as a covariate alongside sociodemographic predictors) was not pre-specified and proved statistically inadmissible owing to quasi-complete separation and an events-per-variable ratio below 3; an indirect adjustment argument is therefore reported in Section 3.4 as a sensitivity consideration.

### 2.8. Ethical Considerations, Consent, and Data Protection

The conduct of this retrospective study on emergency hospitalizations for SUDs was grounded in respect for individual rights and the classical principles of bioethics (respect for autonomy, beneficence, non-maleficence, and justice), in accordance with the international standards governing medical research on human subjects (Declaration of Helsinki, Belmont Report, Nuremberg Code). At the national level, conduct was aligned with the applicable legal framework for medical services and patient rights (Law No. 95/2006 and Law No. 46/2003, with emphasis on confidentiality and the protection of personal data), as well as the relevant professional standards (Romanian College of Physicians and Romanian Society of Psychiatry) for clinical research ethics.

Given the retrospective nature of the design, the analysis used exclusively information already documented in the clinical records, without introducing additional procedures or interventions beyond the standard of care, thus circumscribing a minimal risk profile for patients. Accordingly, any recommendation derived from the results of the analysis was conceptualized as a measure to optimize the quality of care and to reinforce clinical safety, not as a substitute for prospective validation.

The ethical governance component was ensured by obtaining institutional approval before data extraction and analysis: the protocol was submitted to the Ethics Committee of Psychiatric Hospital “Elisabeta Doamna”, and the approval process included methodological justification, a description of the collection/processing workflow, and measures to reduce risks to confidentiality and information security. Data protection was operationalized through an identifiability minimization strategy and strict access control. Extracted data were used exclusively for scientific purposes, with access limited to the directly involved team; analytical datasets were coded by the assignment of a numeric ID, without retention of direct identifiers (name, Personal Identification Number, address). In compliance with EU Regulation 2016/679 (GDPR) and its national implementing legislation (Law No. 190/2018), technical and organizational measures were implemented to secure electronic access and to archive original documents, to prevent any unauthorized disclosure. The database was password-protected, and files exported for analysis were stored on secure/encrypted media; anonymization was completed before statistical analysis, so that there was no moment of simultaneous access to clinical data and identifying elements.

Concerning informed consent, the study utilized historical data; patients had signed informed consent at admission that included agreement for the use of their data in subsequent analyses for scientific purposes, with documents retained in the hospital archive in accordance with legal requirements. In the reporting of results, the principle of non-identification was also maintained by presenting aggregated information (percentages, age intervals, categories) to reduce the risk of indirect re-identification.

## 3. Results

### 3.1. Cohort Description and Temporal Distribution

**Figure 1:**
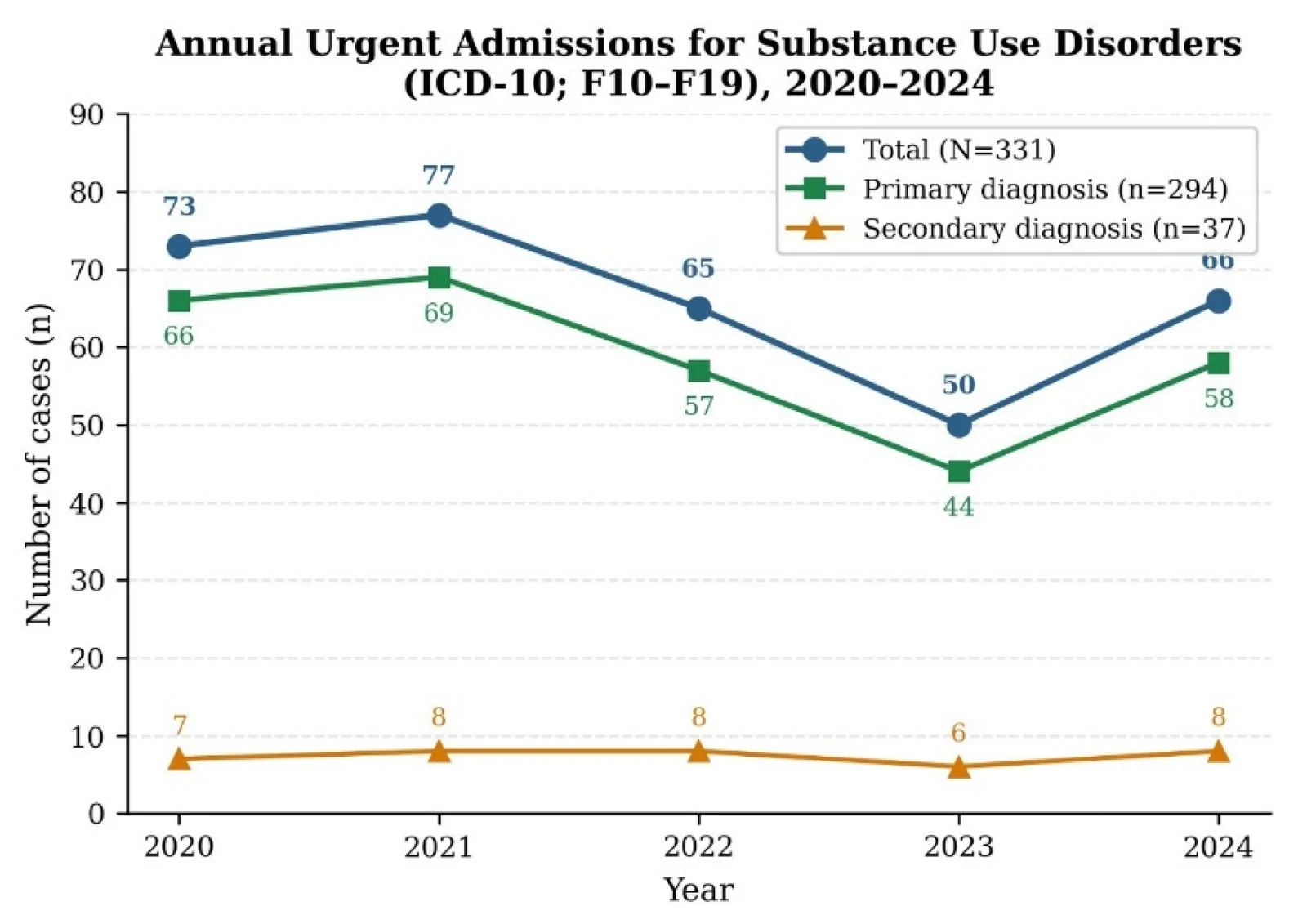
Annual volume of emergency admissions for substance use disorders (ICD-10; F10–F19), 2020–2024. Line chart with three series: total cohort, primary diagnosis cases, and secondary diagnosis cases. Values represent the annual number of admissions (n).

Over the five-year study period (January 2020–December 2024), a total of *N* = 331 emergency admissions meeting the eligibility criteria were identified, of which 294 (88.8%) carried an ICD-10 F10–F19 code as the primary diagnosis and 37 (11.2%) as a secondary diagnosis. The annual volume of emergency admissions (Figure 1). remained relatively stable across the study period, without a pronounced secular trend (range: 50–77 admissions per year).

### 3.2. Sociodemographic and Clinical Profile of the Cohort

A note on group-level baseline balance is warranted. Because treatment strategy was not randomized and was not a stratification factor in the original data extraction protocol, disaggregated baseline characteristics stratified by strategy (pharmacotherapy-alone vs. integrated) could not be reconstructed retrospectively from the routinely documented variables with the granularity needed to support formal balance testing; the pronounced group-size imbalance (n = 38 vs. n = 293) would additionally produce cells with 0–2 observations across several categorical strata, making chi-square and Fisher exact tests of balance uninformative. The cohort-level profile presented in Table 6 therefore describes the baseline composition of the analytic sample as a whole; the potential imbalance between treatment groups on documented and unmeasured characteristics—most notably initial symptom severity, which was captured only qualitatively in the clinical documentation and could not be operationalized as a structured baseline variable—is discussed as a confounding-by-indication limitation in Section 4.3.

**Figure 2:**
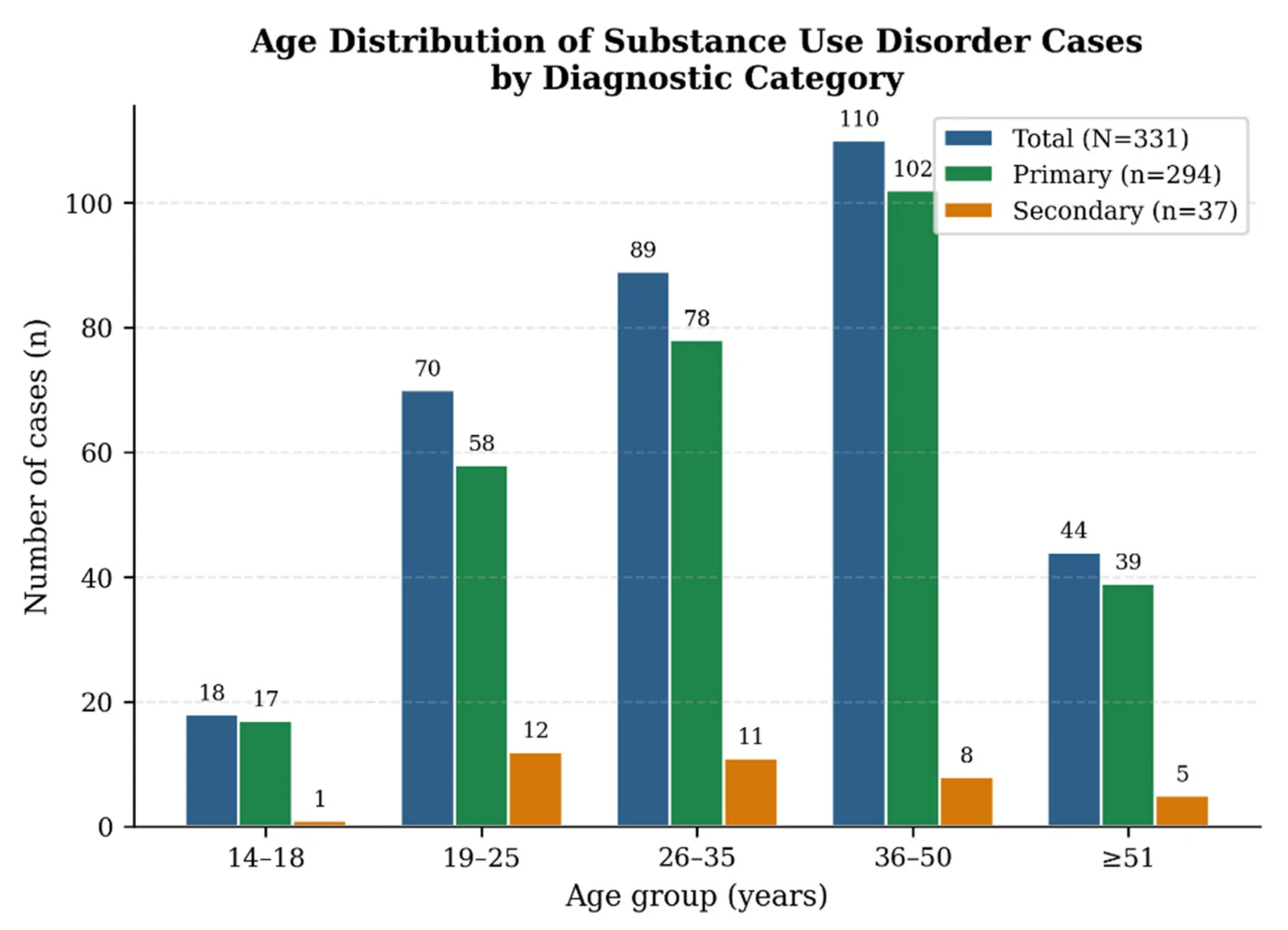
Age distribution of substance use disorder cases by diagnostic category (primary + secondary diagnosis). Grouped bar chart comparing absolute and percentage distributions across age groups.

**Figure 3:**
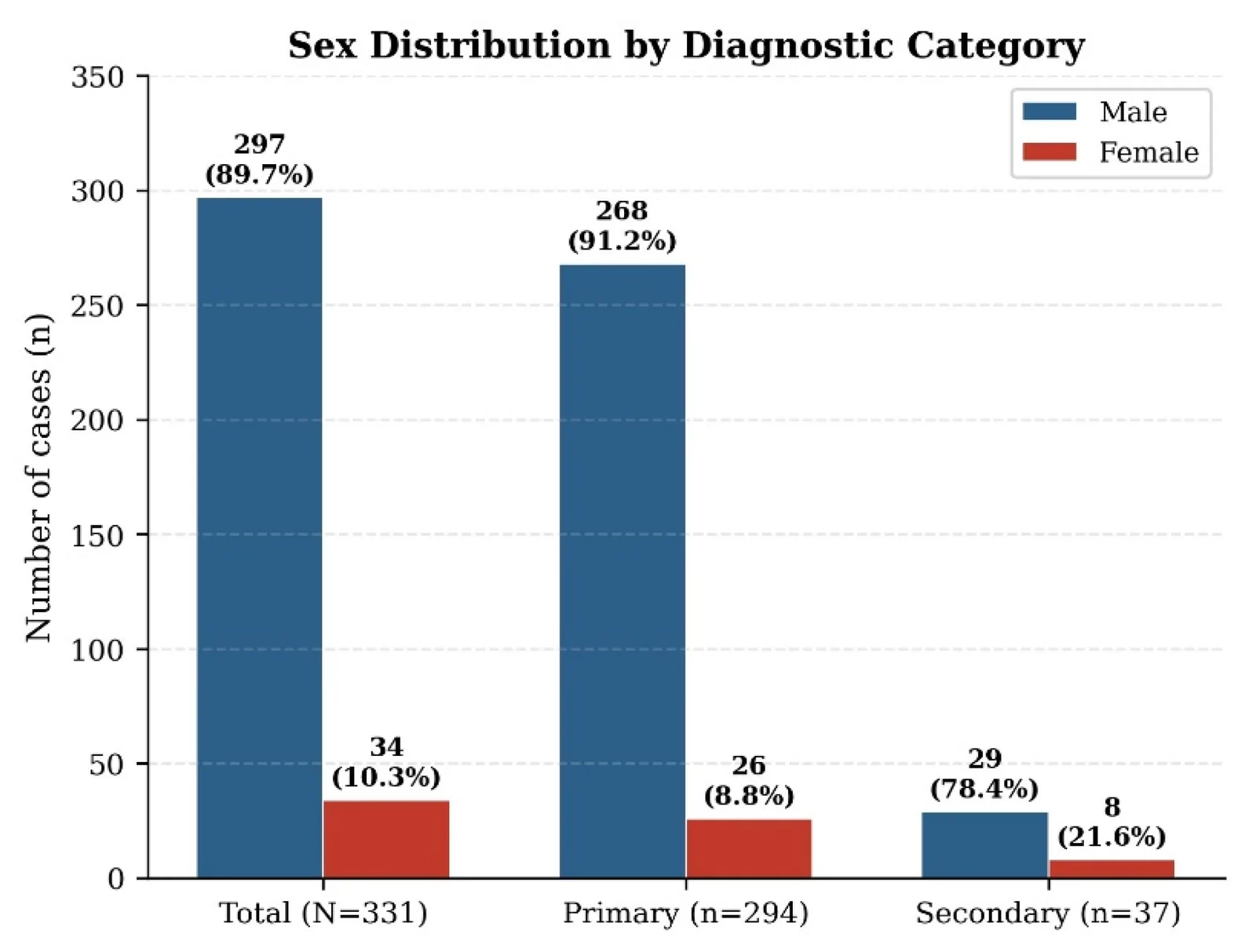
Sex distribution across the total cohort and by diagnostic category (primary vs. secondary diagnosis). Grouped bar chart; each bar indicates the number of cases (n) and the corresponding percentage (%).

The sociodemographic characteristics and initial clinical profile of the study cohort are summarized in Table 6. The sample exhibited a predominantly male composition (n = 297; 89.7%), with a majority urban provenance (n = 189; 57.1%). The age distribution (Figure 2) was concentrated in the young-adult to middle-aged range, with the 36–50 age group being the most represented (n = 110; 33.2%), followed by 26–35 years (n = 89; 26.9%), 19–25 years (n = 70; 21.1%), ≥51 years (n = 44; 13.3%), and 14–18 years (n = 18; 5.4%) (Figure 3). Regarding occupational status, the majority of patients were registered as unemployed (n = 260; 78.5%), followed by employed individuals (n = 28; 8.5%) and old-age pensioners (n = 28; 8.5%); students represented 3.9% (n = 13) and disability pensioners 0.6% (n = 2). Clinically, the predominant primary diagnosis was alcohol dependence syndrome (F10.2; n = 192/294; 65.3%), followed by polysubstance use disorders (F19.*; n = 82/294; 27.9%). Among secondary diagnoses (n = 37), the most frequent categories were stimulants (F15.*; n = 9; 24.3%), volatile solvents (F18.*; n = 9; 24.3%), and cannabinoids (F12.*; n = 8; 21.6%). Psychiatric and/or somatic comorbidities were documented in 152 patients (45.9%).

### 3.3. Clinical Outcome at Discharge (GOSS)

The primary outcome, clinical status at discharge, was classified according to the GOSS framework into three categories: improved, stationary, and worsened. The overall discharge profile was predominantly favorable: 317 of 331 patients (95.8%) were classified as improved, 8 (2.4%) as stationery, and 6 (1.8%) as worsened. While suboptimal outcomes (stationary + worsened; n = 14; 4.2%) were numerically rare, they are clinically significant given their disproportionate association with risk concentration, management complexity, and resource consumption.

**Figure 4:**
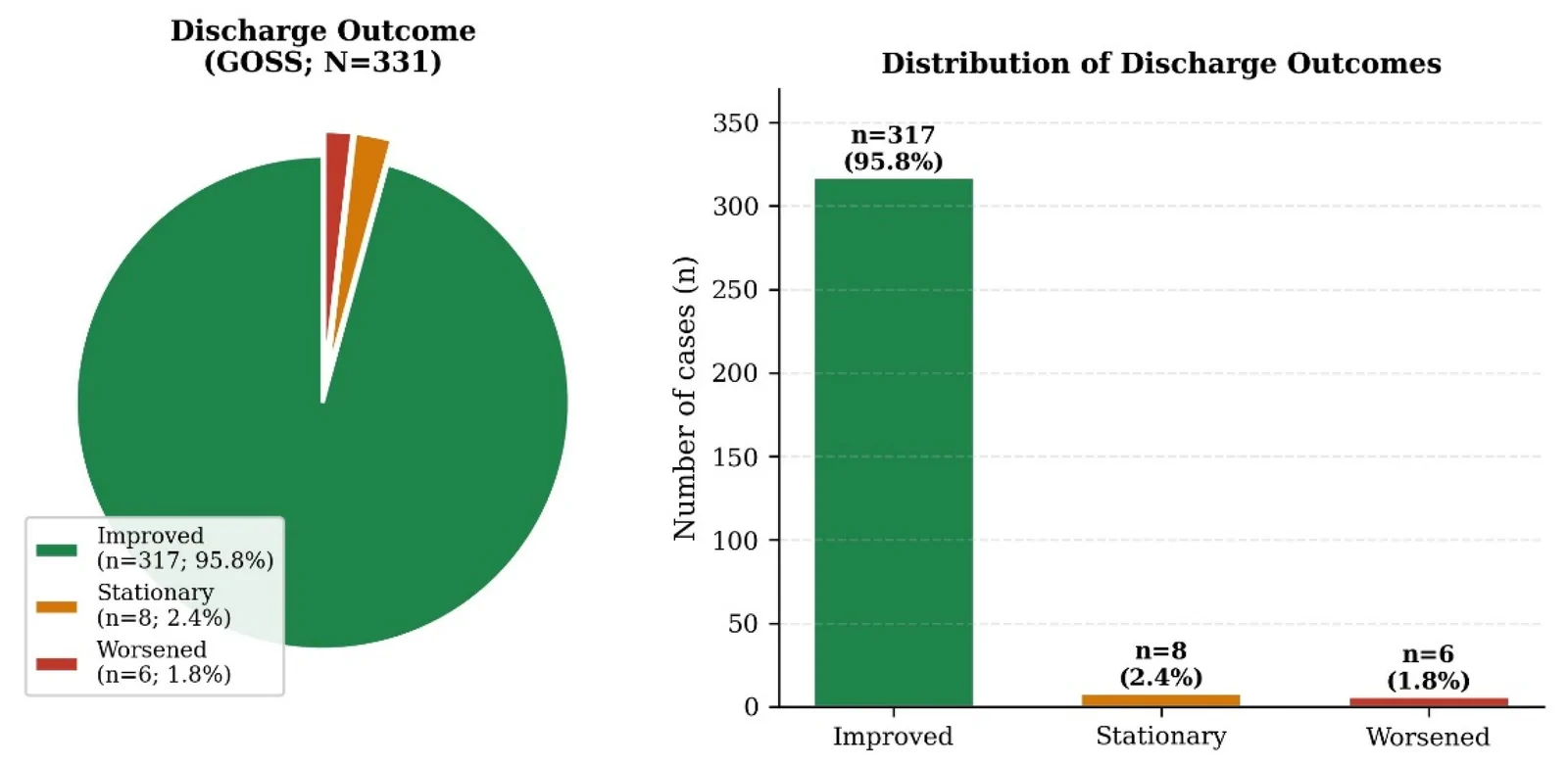
Overall distribution of discharge outcomes (GOSS) in the study cohort (N = 331). Pie or bar chart showing the proportions of improved (95.8%), stationery (2.4%), and worsened (1.8%) outcomes.

One-way ANOVA indicated statistically significant annual variation in therapeutic success rates across the five study years (F = 4.32; *p* = 0.008) (Figure 4).

### 3.4. Primary Analysis (H_01_/H_11_): Association Between Therapeutic Strategy and Improvement at Discharge

The integrated therapeutic strategy, as operationalized in Section 2.1, was the dominant management approach, applied in 293 of 331 cases (88.5%). The pharmacotherapy-alone strategy was applied in the remaining 38 cases (11.5%).

The pronounced group-size imbalance (38 vs. 293; 11.5% vs. 88.5%) is a direct reflection of real-world clinical practice in this emergency psychiatric setting, where integrated care constituted the routine management pathway and pharmacotherapy alone was the exception rather than the norm (see Section 2.1 for the clinical determinants of allocation). This naturalistic asymmetry has identifiable statistical consequences: (i) the small pharmacotherapy-alone stratum reduces the precision of between-group effect estimates, as reflected in the wide confidence interval of the primary OR (5.62–56.91); (ii) several cells in the 2 × 3 contingency table contain fewer than five observations, precluding the use of chi-square tests and motivating the selection of Fisher’s exact test as the principal inferential method; and (iii) Wilson confidence intervals were applied to individual proportions, and the Newcombe–Wilson method to the absolute risk difference, given that both methods perform well with extreme proportions and small denominators. These analytical choices do not eliminate the imprecision inherent in a small comparator group but ensure that the reported estimates and *p*-values are methodologically appropriate for the observed data structure.

The distribution of discharge outcomes across the two strategies is presented in Table 7.

Descriptively, the improvement rate was markedly higher in the integrated strategy group (288/293; 98.3%; 95% CI Wilson: 96.1–99.3) compared to the pharmacotherapy-alone group (29/38; 76.3%; 95% CI Wilson: 60.8–87.0), yielding an absolute difference of +21.98 percentage points (pp) (95% CI Newcombe–Wilson: 9.06–38.48) (Table 8). Conversely, suboptimal outcomes (stationary/worsened) concentrated disproportionately in the pharmacotherapy-alone group (9/38; 23.7%) versus the integrated group (5/293; 1.7%), with a particularly notable difference for the worsened category (10.5% vs. 0.7%).

**Figure 5:**
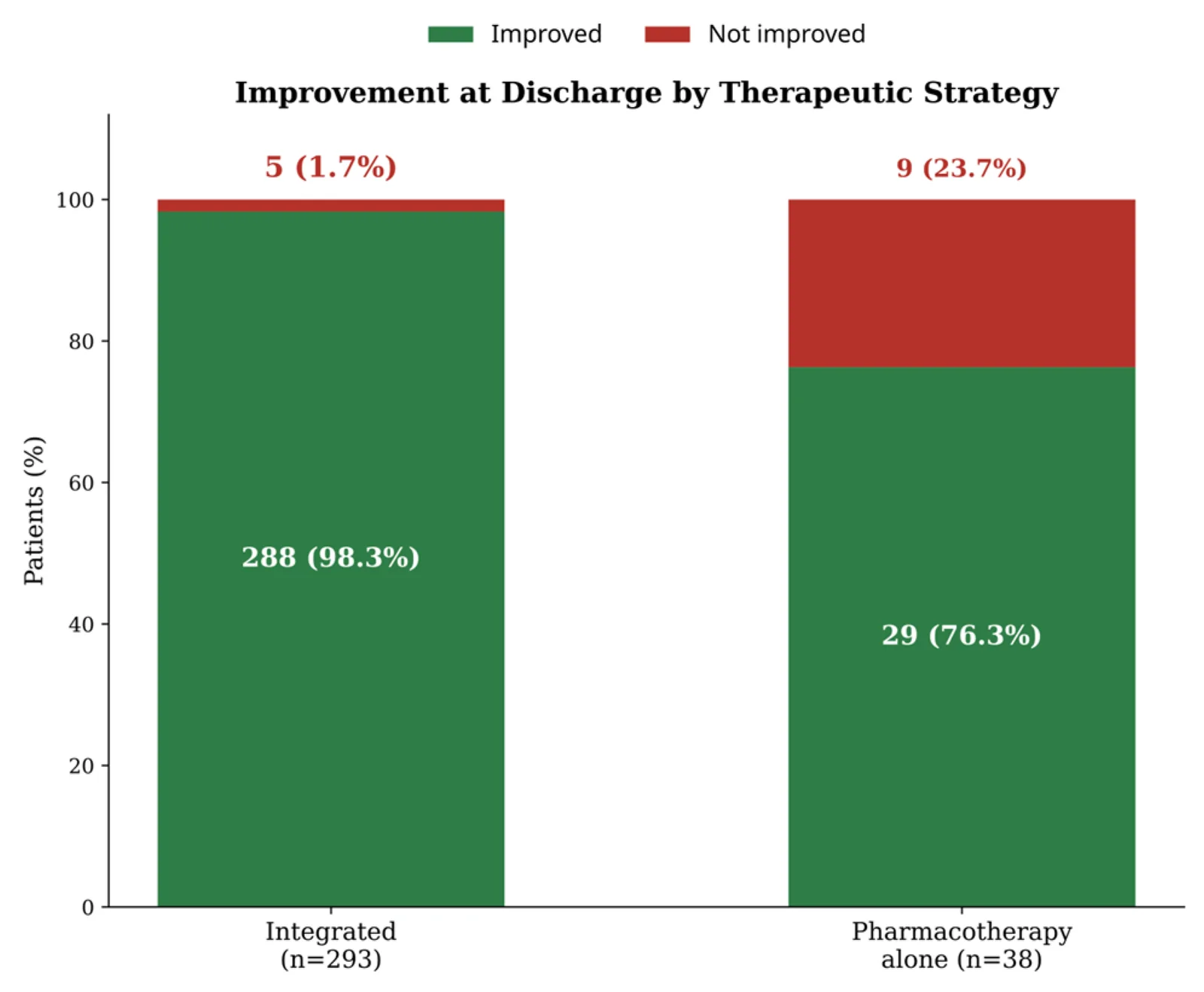
Improvement at discharge by therapeutic strategy (primary endpoint; binary recoding: improved vs. not improved). 100% stacked bar chart comparing the proportions of improved versus not improved patients between the integrated strategy (n = 293) and pharmacotherapy alone (n = 38).

**Figure 6:**
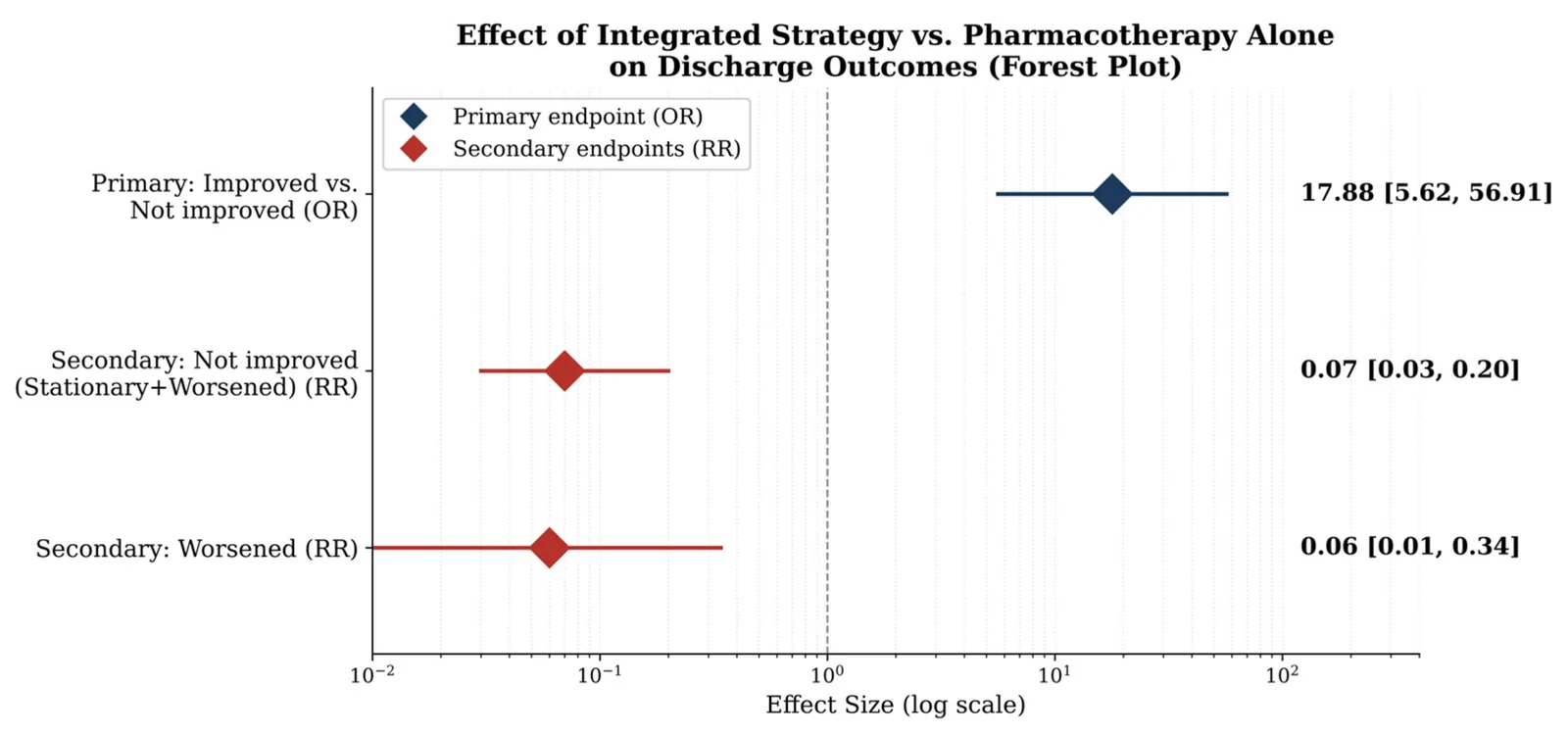
Forest plot summarizing the effect of the integrated strategy versus pharmacotherapy alone on discharge outcomes. The horizontal axis represents effect size on a logarithmic scale; the dashed vertical line marks the null value (1.0). The black diamond corresponds to the primary endpoint (OR for improvement); red diamonds correspond to secondary endpoints (RR for non-improvement and worsening). Values to the right of 1.0 indicate higher odds of improvement; values to the left of 1.0 for secondary endpoints indicate risk reduction.

Inferentially, the association between therapeutic strategy and improvement at discharge was strong and convergent across all endpoints (Table 9). For the primary endpoint (improved vs. not improved), the odds of improvement were substantially higher under the integrated strategy (OR = 17.88; 95% CI: 5.62–56.91; Fisher’s exact test, *p* < 0.001). The integrated strategy was also associated with a marked reduction in the risk of non-improvement (RR = 0.07; 95% CI: 0.03–0.20; *p* < 0.001) and worsening (RR = 0.06; 95% CI: 0.01–0.34; *p* = 0.002). The Cramér V/φ coefficient was approximately 0.35, indicating a moderate-to-large association at the cohort level. In clinically translatable terms, the estimated number needed to treat (NNT) was approximately 5 (95% CI: 3–12).These effect measures are illustrated in Figure 5, with the corresponding absolute outcome proportions shown in Figure 6.

A formal multivariable model including treatment strategy as a covariate was not estimable: the 14 suboptimal events are concentrated in the pharmacotherapy-alone group (9/38 vs. 5/293), producing quasi-complete separation, and the resulting events-per-variable ratio (~2.8 with five predictors) falls below conventional stability thresholds (Figure 5).

As an alternative, we note that the two strongest measured confounders of the strategy–outcome relationship—age ≥ 51 years (adjusted OR = 4.2) and unemployment (adjusted OR = 3.1)—have effect sizes substantially smaller than the unadjusted strategy association (OR = 17.88). Applying the logic of Cornfield’s inequality, an unmeasured confounder capable of entirely explaining the observed association would need to be both far more prevalent in the pharmacotherapy-alone group and far more strongly associated with suboptimal outcome than any documented variable in this dataset. While residual confounding almost certainly attenuates the true effect size, the direction and clinical relevance of the association are unlikely to be explained away entirely by measured or plausible unmeasured confounders. The unadjusted OR of 17.88 should therefore be interpreted as an upper bound; the true adjusted effect, if estimable, would plausibly be smaller but is unlikely to cross the null (Figure 6).

### 3.5. Secondary Analyses

#### 3.5.1. Length of Stay and Probability of Improvement (H_02_/H_12_)

Length of stay (LOS) was treated as a pragmatic indicator of initial severity and management complexity. The distribution of discharge outcomes across LOS categories is presented in Table 10. The Pearson χ^2^ test on the 4 × 3 contingency table indicated a statistically significant association between LOS and discharge outcome (χ^2^(6) = 23.42; *p* = 0.0007; Cramér’s V = 0.19), confirmed by an exact RxC test with Monte Carlo simulation (500,000 replications; *p*_MC = 0.00435 for χ^2^ and *p*_MC = 0.00336 for the likelihood ratio G^2^).

**Figure 7:**
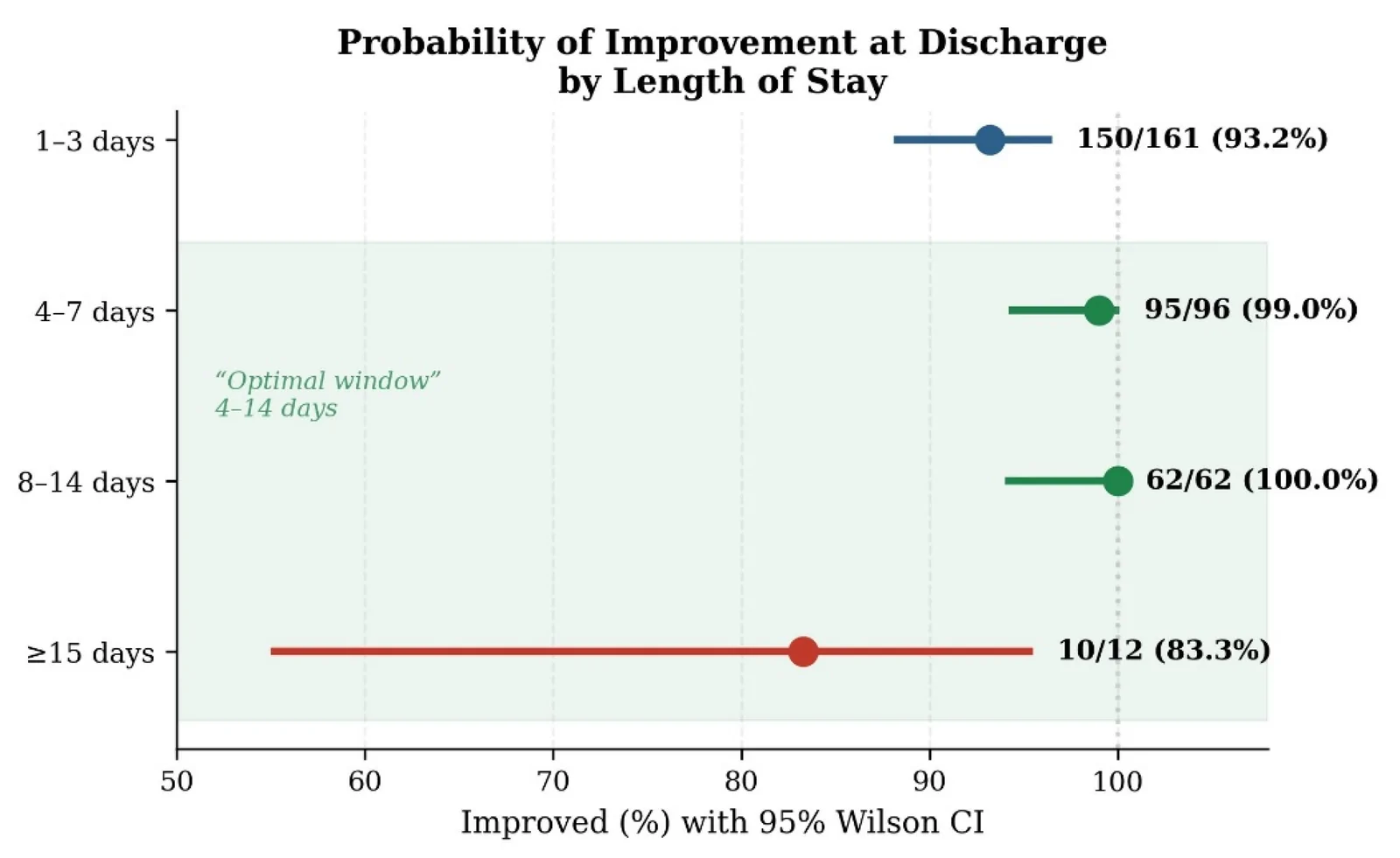
Probability of improvement at discharge by length of stay category (95% CI Wilson). Dot plot with horizontal confidence interval bars for each LOS category (1–3 days, 4–7 days, 8–14 days, ≥15 days), highlighting the 4–14 day window as the zone associated with the highest improvement probability.

A clear gradient emerged: the 4–14 day window was associated with the highest probability of improvement. Specifically, the 8–14 day category achieved a 100% improvement rate (62/62), followed by 4–7 days (95/96; 99.0%), while short stays (1–3 days; 150/161; 93.2%) and prolonged hospitalizations (≥15 days; 10/12; 83.3%) yielded lower improvement rates and higher uncertainty. When comparing the 4–14 day “optimal window” against remaining durations, the absolute difference in improvement was +6.88 pp, with a marked reduction in the odds of non-improvement (OR for non-improvement ≈ 0.078), consistent with a pragmatic “optimal hospitalization window” associated with the highest probability of improvement and the lowest risk of worsening (Figure 7).

#### 3.5.2. Comorbidities and Risk of Suboptimal Response (H_03_/H_13_)

Among the 152 patients with at least one documented comorbidity (45.9% of the cohort), the comorbidity profile was dominated by somatic conditions (68% of comorbid cases), most frequently alcoholic liver disease (43%), chronic ischemic heart disease (22%), and chronic viral infections (HIV/HCV; 18%). Psychiatric comorbidities accounted for the remaining 32% of comorbid cases, led by major depressive disorder (61%) and borderline personality disorder (29%).

The presence of documented comorbidities (psychiatric and/or somatic) was associated with a numerically higher rate of suboptimal response at discharge. Among patients with comorbidities, 9 of 152 (5.9%; 95% CI Wilson: 3.1–10.9) experienced a suboptimal outcome, compared with 5 of 179 (2.8%; 95% CI Wilson: 1.2–6.4) among those without comorbidities (Table 11). The absolute risk difference was +3.13 pp (95% CI Newcombe–Wilson: −1.40 to 8.33), suggesting an increased probability of a stationary/worsened outcome in the presence of comorbidities, albeit with substantial uncertainty (the confidence interval includes zero). In relative terms, the effect estimates pointed in the direction of the alternative hypothesis (RR = 2.12; 95% CI: 0.73–6.19; OR = 2.19; 95% CI: 0.72–6.68), but Fisher’s exact test did not support a robust association at the conventional threshold (*p* = 0.180). These results are interpreted as a signal/trend rather than definitive evidence, warranting further investigation in larger samples with more granular comorbidity classification.

#### 3.5.3. Sociodemographic Predictors of Suboptimal Response (H_04_/H_14_)

Chi-square goodness-of-fit analyses confirmed significant deviations from expected uniform distributions across demographic variables: age groups (χ^2^ = 72.18; df = 4; *p* < 0.0001), area of residence (χ^2^ = 6.74; df = 1; *p* = 0.009; urban overrepresentation by +7.3%), and occupational status (χ^2^ = 1204.2; df = 4; *p* < 0.0001; unemployed group overrepresented by +194% vs. expected). Cramér’s V coefficients indicated significant correlations between suboptimal response and age ≥ 51 years (V = 0.21; *p* = 0.002) and unemployment (V = 0.16; *p* = 0.018), while rural residence did not reach statistical significance (V = 0.09; *p* = 0.12).

Among the 260 unemployed patients, 239 (92%) lacked health insurance, and only 31 (12%) had documented access to post-discharge rehabilitation programs.

An exploratory binary logistic regression model was constructed with suboptimal outcome (stationary/worsened = 1; improved = 0) as the dependent variable. Given the small number of events (n = 14; events-per-variable ratio ~3.5), this analysis is hypothesis-generating; among the four candidate covariates, only age ≥ 51 years and unemployment—both significant in prior bivariate testing—are interpreted as candidate risk markers, while the remaining covariates are reported for transparency of the full model. The results are summarized in Table 12.

**Figure 8:**
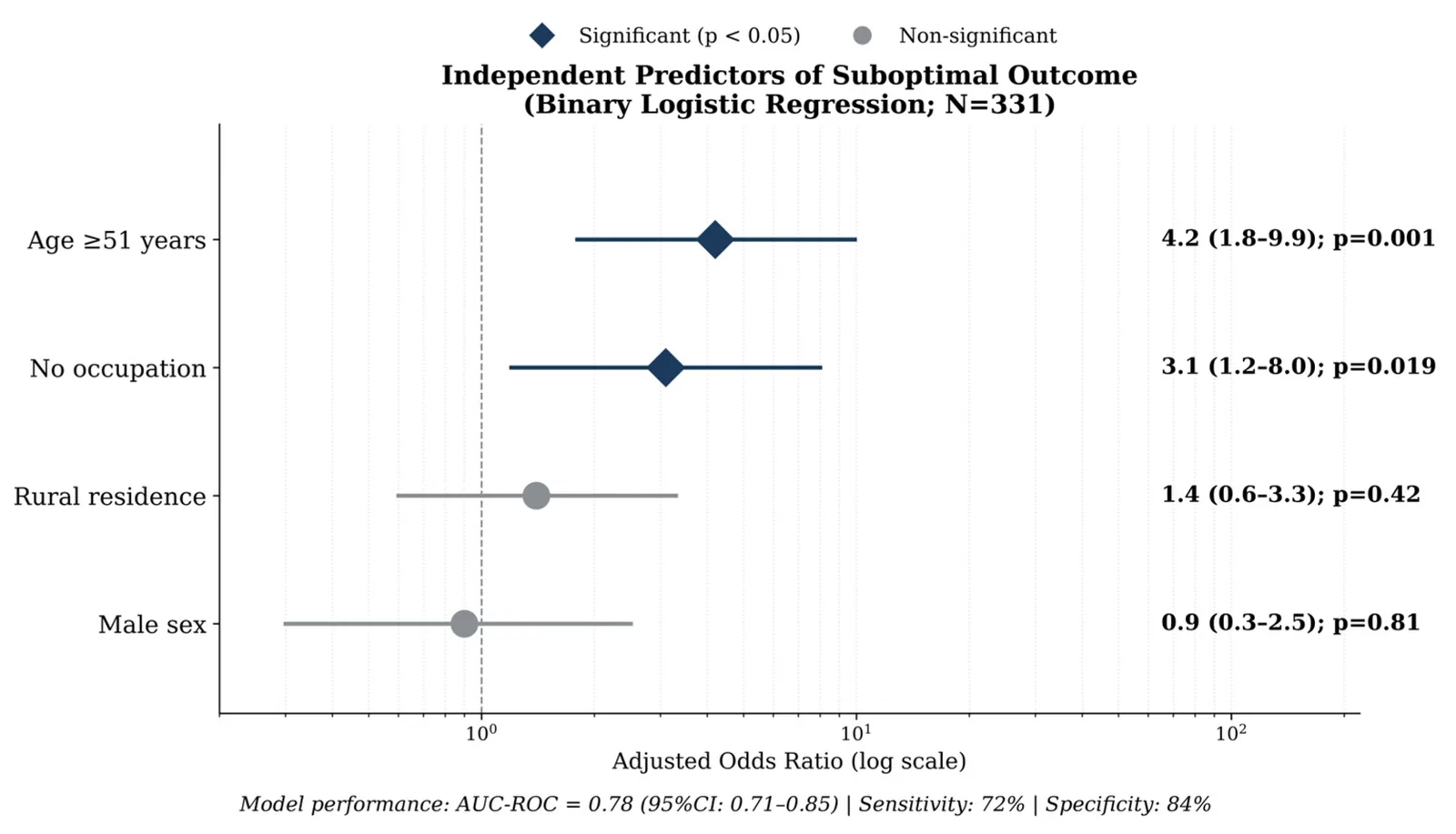
Forest plot of adjusted odds ratios for independent predictors of suboptimal discharge outcome (binary logistic regression). Vertical reference line at OR = 1 indicates no association (null effect). Points to the right indicate increased odds of suboptimal outcome. Horizontal bar length reflects the precision of the estimate (95% CI width).

Age ≥ 51 years was associated with a 4.2-fold increased odds of suboptimal outcome (adjusted OR = 4.2; 95% CI: 1.8–9.9; *p* = 0.001), and unemployment was associated with a 3.1-fold increase (adjusted OR = 3.1; 95% CI: 1.2–8.0; *p* = 0.019). Rural residence (adjusted OR = 1.4; 95% CI: 0.6–3.3; *p* = 0.42) and male sex (adjusted OR = 0.9; 95% CI: 0.3–2.5; *p* = 0.81) were not significant independent predictors. The discriminatory performance of the model was acceptable (AUC-ROC = 0.78; 95% CI: 0.71–0.85), with a sensitivity of 72% and specificity of 84%, suggesting practical utility for risk stratification. Consistent with the exploratory nature of this analysis, the strong class imbalance (few events) may amplify coefficient instability and widen confidence intervals; these estimates therefore require prospective external validation on independent samples before any inferential or clinical use (Figure 8).

### 3.6. Summary of Key Findings

In summary, the results converge on the following principal findings: (i) the immediate prognosis at discharge was favorable for the vast majority of patients (95.8% improved); (ii) the integrated therapeutic strategy was associated with a substantially higher probability of improvement compared to pharmacotherapy alone (OR = 17.88; 95% CI: 5.62–56.91; *p* < 0.001), with an absolute difference of +22 pp and an NNT of approximately 5 (95% CI: 3–12), although the wide confidence interval reflects considerable uncertainty around the point estimate; (iii) a pragmatically “optimal” hospitalization window of 4–14 days was identified, with the highest improvement rates and lowest suboptimal outcomes; (iv) comorbidities showed a non-significant trend toward increased suboptimal response (*p* = 0.180); and (v) suboptimal outcomes, although rare (4.2%), were concentrated in identifiable subgroups—patients aged ≥51 years and unemployed individuals, providing an operational basis for risk stratification, monitoring intensity, and safe discharge planning.

## 4. Discussion

### 4.1. Summary of Principal Findings

This single-center, retrospective, observational study evaluated five years (2020–2024) of emergency psychiatric admissions for substance use disorders (SUDs; ICD-10 F10–F19) at a Romanian psychiatric hospital, focusing on the comparative effectiveness of two management strategies: pharmacotherapy alone versus an integrated therapeutic approach. The principal findings can be summarized along four convergent axes.

First, regarding the primary outcome, the integrated strategy, defined as pharmacotherapy combined with at least one non-pharmacological component and/or clinically relevant paraclinical assessment, was associated with a substantially higher probability of clinical improvement at discharge compared with pharmacotherapy alone (98.3% vs. 76.3%; OR = 17.88; 95% CI: 5.62–56.91; *p* < 0.001). The absolute risk difference was approximately 22 percentage points (95% CI: 9.06–38.48, Newcombe–Wilson method), corresponding to an estimated number needed to treat (NNT) of approximately 5 (95% CI: 3–12). Complementarily, integrated management was associated with a marked reduction in the risk of non-improvement (RR = 0.07; 95% CI: 0.03–0.20) and worsening (RR = 0.06; 95% CI: 0.01–0.34). These effect measures are synthesized in the forest plot presented in Figure 6 (Louie et al., 2020; Ray et al., 2020).

Second, secondary analyses examining the relationship between length of stay and discharge outcome (Table 10; Figure 8) identified a pragmatic “optimal window” of 4–14 days, during which improvement rates exceeded 99% (with 100% in the 8–14-day interval). Very short admissions (1–3 days) were associated with a suboptimal response rate of 6.8%, reflecting premature discharge and potential underestimation of initial severity, while prolonged hospitalizations (≥15 days) were associated with a worsening rate of 16.7%, likely attributable to unrecognized somatic comorbidities and refractory withdrawal (Soyka et al., 2017; Wolf et al., 2020).

Third, although comorbidities showed a trend toward a higher risk of suboptimal response (5.9% vs. 2.8%), this difference did not reach statistical significance in the exact test (*p* = 0.180) and should be interpreted as a clinical signal rather than a definitive association.

Fourth, exploratory binary logistic regression modeling (Table 12; Figure 8) identified two candidate independent predictors of suboptimal outcome at discharge: age ≥ 51 years (adjusted OR = 4.2; 95% CI: 1.8–9.9; *p* = 0.001) and unemployment (adjusted OR = 3.1; 95% CI: 1.2–8.0; *p* = 0.019). Rural residence and male sex did not retain significance after adjustment. The model exhibited acceptable discriminative performance (AUC-ROC = 0.78; 95% CI: 0.71–0.85), with a sensitivity of 72% and a specificity of 84%, supporting its utility primarily for risk exclusion (NPV = 98.5%) in the context of a low event prevalence (4.2%) (Ranganathan et al., 2017; Soyka et al., 2017).

### 4.2. Interpretation and Contextualization of Findings

#### 4.2.1. Integrated Strategy and Discharge Improvement

The **principal finding**—that integrated management was associated with a substantially higher probability of improvement at discharge—is consistent with the conceptual framework underpinning current international guidelines for SUD treatment. The WHO International Standards for Drug Use Disorder Treatment (*International standards for the treatment of drug use disorders*, n.d.), the NICE guidelines (NICE, 2011), and the APA Practice Guideline for Alcohol Use Disorder (Reus et al., 2018) consistently advocate for multimodal interventions that combine pharmacotherapy with psychosocial support, structured monitoring, and continuity of care. In this study, the integrated strategy encompassed not only pharmacological agents but also crisis-supportive psychotherapy, motivational counseling, psychoeducation, nutritional assessments, and comprehensive paraclinical investigations, thereby approximating a structured biopsychosocial model within the constraints of an emergency setting.

The magnitude of the observed association (OR = 17.88) warrants cautious interpretation. The direction of the effect and its statistical significance are consistent across all endpoints (Table 8), but the wide confidence interval (5.62–56.91) means the true effect size is compatible with any value from a moderate association (OR ≈ 6) to a very large one (OR > 50). This imprecision is a direct consequence of the small comparator group (n = 38) and the rarity of suboptimal events (n = 14; 4.2%), and the point estimate should be read as an upper bound rather than as a stable effect size—particularly given the residual confounding discussed in Section 4.3. The observed magnitude is consistent with a “therapeutic package” effect, whereby the association may reflect the cumulative contribution of multiple complementary interventions rather than any single component, an interpretation compatible with recent meta-analytic evidence on combined pharmacological and psychosocial approaches (Ray et al., 2020). The Cramér V coefficient of approximately 0.35 indicates a moderate-to-large association at the cohort level, as visualized in the absolute impact comparison (Figure 7; Table 9).

Even under the most conservative interpretation—assuming that a non-trivial portion of the observed association is attributable to residual confounding by indication (Section 4.3)—the direction and clinical relevance of the finding are unlikely to reverse; the point estimate should nonetheless be read as an upper bound rather than as a causal effect size, and the true effect is plausibly smaller than 17.88.

The NNT of approximately 5 (95% CI: 3–12) provides a rough order-of-magnitude indicator of the potential clinical benefit: under the observed proportions, for every 5 patients managed with an integrated rather than pharmacotherapy-alone strategy, one additional patient would be expected to improve at discharge. However, this estimate inherits the same imprecision and vulnerability to confounding as the underlying OR; it should be interpreted as a hypothesis-generating approximation derived from an observational, non-randomized comparison rather than as a definitive benchmark for resource allocation.

#### 4.2.2. Length of Stay and the Optimal Therapeutic Window

The identification of a 4–14-day hospitalization window associated with maximal improvement rates (Table 10) has practical relevance for discharge planning and resource utilization. This finding aligns with the clinical pharmacology of withdrawal management, particularly for alcohol dependence (the dominant diagnosis in this cohort at 65.3%), where the typical course of acute withdrawal spans 5–10 days and pharmacological titration (benzodiazepines, antipsychotics, hepatoprotective agents) requires iterative adjustment and monitoring (Soyka et al., 2017; Tiglao et al., 2021; Wolf et al., 2020).

Very short admissions (1–3 days), which accounted for nearly half the cohort (n = 161; 48.6%), were associated with a higher proportion of stationary and worsened outcomes. This pattern may reflect several non-mutually exclusive mechanisms: premature self-discharge, underestimation of withdrawal severity at admission, or insufficient time for non-pharmacological interventions to exert their effect. Conversely, prolonged hospitalizations (≥15 days) were associated with worsening in 16.7% of cases, likely capturing patients with complex comorbidities (hepatopathy, refractory withdrawal, somatic decompensation) who required extended monitoring and therapeutic escalation. Notably, 75% of patients hospitalized for more than two weeks were of rural origin, suggesting that delayed initial intervention and limited access to pre-hospital services may contribute to both prolonged stays and unfavorable outcomes.

The association between length of stay and discharge outcome (χ^2^(6) = 23.42; *p* = 0.0007; Cramér V = 0.19) must, however, be interpreted cautiously given the possibility of reverse causality: severity may prolong hospitalization, and hospitalization may facilitate stabilization, creating a bidirectional relationship that limits causal inference from cross-sectional comparisons (Tiglao et al., 2021).

#### 4.2.3. Sociodemographic Predictors of Suboptimal Response

The finding that age ≥ 51 years and unemployment emerged as independent predictors of suboptimal response (Table 12; Table 6) has both biological and social plausibility. Older patients with SUDs frequently present with accumulated organ damage (hepatic, neurological, cardiovascular), altered drug metabolism, higher rates of polypharmacy, and diminished physiological reserve for withdrawal, factors that independently reduce the probability of rapid clinical stabilization (Soyka et al., 2017). In this cohort, the adjusted OR of 4.2 for age ≥ 51 years indicates that the odds of a suboptimal outcome were more than four times higher in this subgroup, after controlling for occupational status, residence, and sex.

Unemployment, as the second independent predictor (adjusted OR = 3.1), likely functions as a proxy for a constellation of psychosocial vulnerabilities: lack of health insurance coverage, absence of structured daily routines, limited social support networks, and reduced access to post-discharge rehabilitation programs. In this cohort, 92% of unemployed patients lacked health insurance, and only 12% had documented access to post-discharge rehabilitation, structural barriers that perpetuate the cycle of relapse and re-admission. These findings resonate with international evidence linking socioeconomic deprivation to poorer SUD treatment outcomes and higher dropout rates (Harris et al., 2019; Rehm & Shield, 2019b).

Neither rural residence (adjusted OR = 1.4; *p* = 0.42) nor male sex (adjusted OR = 0.9; *p* = 0.81) retained significance in the multivariate model, although the cohort’s strong male predominance (89.7%) and the urban majority (57.1%) limit statistical power to detect modest effects for these variables.

#### 4.2.4. Cohort Profile in Regional and International Context

The demographic and nosological profile of this cohort, predominantly male (89.7%), urban (57.1%), young-to-middle-aged (peak 36–50 years), unemployed (78.5%), and with alcohol dependence as the leading primary diagnosis (65.3%), is broadly consistent with European epidemiological data on emergency SUD presentations (Degenhardt et al., 2018; European Union Drugs Agency, 2024; Rehm & Shield, 2019a). The 2024 European Drug Report confirms that alcohol-related disorders remain the primary driver of SUD-associated hospital contacts across the European Union, with polysubstance use (F19.* codes) constituting a growing secondary pattern, as reflected in the present cohort (27.9% of primary diagnoses) (European Union Drugs Agency, 2024).

The widespread unemployment and its link to negative outcomes highlight the connection between addiction and social exclusion, a factor that is especially strong in southeastern Romania, where addiction treatment infrastructure is limited and community-based rehabilitation efforts are underdeveloped. These contextual factors, while increasing the ecological validity of the study, also limit its generalizability to settings with different service setups and population characteristics.

#### 4.2.5. Pandemic and Post-Pandemic Dynamics (2020–2024)

The study period encompasses the COVID-19 pandemic and its aftermath, which introduced significant perturbations to both substance use patterns and healthcare delivery. The observed decline in admission numbers during 2022–2023, followed by a recovery to pre-pandemic levels in 2024, is consistent with international reports of reduced healthcare-seeking behavior during the pandemic, combined with shifts in substance availability and consumption patterns (*Global status report on alcohol and health 2018*, n.d.; *World drug report 2024*, n.d.). The psychosocial burden associated with COVID-19 extended beyond direct psychiatric presentations, affecting emergency utilization patterns across multiple medical domains and reinforcing the systemic vulnerability of emergency services during this period with psychiatric morbidity documented in a substantial proportion of inpatients across medical specialties, further supporting the integration of psychiatric evaluation into the acute care pathway (L.-A. Moroianu et al., 2022; M. Moroianu et al., 2026; Pâslaru et al., 2025). Annual variation in therapeutic success rates (*F* = 4.32; *p* = 0.008) likely reflects the interplay between institutional capacity (staffing, resource availability) and epidemiological context, reinforcing the vulnerability of acute psychiatric services to systemic healthcare disruptions.

### 4.3. Methodological Limitations and Validity Considerations

The interpretation of these findings must be anchored in the inherent limitations of a retrospective, single-center, observational design based on real-world clinical data.

First, the retrospective design entails structural dependence on the quality and completeness of clinical documentation (General Clinical Observation File and Emergency Registry), with attendant risk of information bias, including misclassification, underreporting, and variability in clinical annotation. This limitation is particularly relevant for variables with reduced granularity in the observation files, such as initial symptom severity and dynamic symptom trajectories, which may function as unmeasured confounders in the relationship between therapeutic strategy and discharge outcome.

Second, the primary outcome (GOSS) was operationalized as a pragmatic, study-specific classification rather than a formally validated instrument, which entails several related constraints. Classification was performed retrospectively from clinical records using operational benchmarks (e.g., “≥50% improvement” for the “Improved” category), whose application may vary with the consistency of clinical descriptions and the criteria implicitly employed in routine practice. Although outcome coding was subject to independent double-entry with discrepancy reconciliation and a 15% source re-verification (Section 2.2), a formal inter-rater agreement coefficient was not quantified, precluding an empirical estimate of classification reliability. Moreover, because treatment strategy was documented in the same records used to derive the outcome, outcome coding could not be blinded to treatment allocation, introducing a potential risk of assessment bias that may operate in the direction of the observed effect. Finally, the frequent dichotomization of the ordinal outcome (“Improved” vs. “Stationary/Worsened”) enhances statistical robustness under conditions of rare events but reduces informational granularity. These considerations warrant caution in interpreting the magnitude of the reported associations and underscore the need for prospective validation using standardized, blinded outcome assessment.

Third, the comparison of therapeutic strategies is vulnerable to confounding by indication. As detailed in Section 2.1, allocation to the integrated versus pharmacotherapy-alone group was not randomized but reflected the treating psychiatrist’s clinical judgment under emergency-specific constraints (initial severity, level of agitation, patient cooperation, and organizational particularities such as resource availability, team composition, and timing of evaluations). Patients selected for integrated care therefore likely differ systematically from those receiving pharmacotherapy alone on both documented and unmeasured characteristics. The direction of the resulting bias cannot be established with certainty from the retrospective data: on one hand, patients perceived as more clinically complex—with somatic or psychiatric comorbidities requiring paraclinical work-up—were more likely to be routed toward the integrated pathway, which would bias the observed effect toward the null (the integrated group would have started at a clinical disadvantage); on the other hand, patients who were uncooperative, agitated, or who presented outside psychology and social-work working hours were more likely to receive pharmacotherapy alone, and since these features also independently predict poorer discharge outcome, this pattern would bias the observed effect away from the null, inflating the apparent superiority of the integrated strategy. A formal group-level baseline balance table (integrated vs. pharmacotherapy-alone stratified on age, sex, area of residence, occupation, primary ICD-10 diagnosis, comorbidities, and length of stay) could not be reconstructed retrospectively from the routine clinical documentation with the granularity required for meaningful between-group testing; readers are referred to the overall cohort profile in Section 3.2 (Table 6) for the sociodemographic and clinical composition of the sample. The multivariable logistic regression reported in Section 3.5.3 (Table 12), which adjusts for age, sex, occupational status, and area of residence, provides a partial mitigation of confounding by indication; however, unmeasured confounders—most notably initial symptom severity, level of agitation, and real-time staff availability—cannot be reconstructed from routine clinical records, so residual confounding remains probable (Ranganathan et al., 2017). The absence of a formal baseline balance table stratified on treatment strategy is itself a limitation of the retrospective design; the cohort-level baseline profile reported in Table 6 is the closest available approximation but does not permit direct assessment of between-group imbalance.

Fourth, the operationalization of the integrated strategy entails compositional heterogeneity: the exposure category encompassed both psychosocial/behavioral interventions and comprehensive paraclinical assessments, which are conceptually distinct components. Because classification was based on retrospective documentation, patients receiving pharmacotherapy plus clinically relevant investigations, but without a documented psychosocial intervention, were included in the integrated group. This broad definition may dilute or conflate the specific contribution of behavioral and psychosocial components to the observed effect, and the estimated association should therefore be interpreted as reflecting a complex, multimodal management pattern rather than the isolated effect of psychosocial interventions. Future prospective studies should disaggregate these components to quantify their independent contributions. Specifically, the non-pharmacological components (crisis-supportive psychotherapy, motivational counselling, psychoeducation, nutritional assessment, and rehabilitation programs) were documented in clinical records as a composite intervention without systematic individual-level flags; combined with the small number of suboptimal events (n = 14), any stratified outcome analysis would yield cells with insufficient power, precluding meaningful statistical inference or clinical interpretation.

Fifth, the rarity of suboptimal events (~4.2%) and the pronounced group-size imbalance (pharmacotherapy alone: n = 38, 11.5%; integrated: n = 293, 88.5%) impose constraints on the stability and precision of effect estimates. This asymmetry is not a sampling artefact but a naturalistic consequence of routine emergency psychiatric practice, where integrated care was the default pathway; artificially equalizing the groups through retrospective matching or oversampling was neither feasible nor methodologically appropriate, as it would have altered the research question from a pragmatic description of care-as-delivered to a simulated experimental comparison. The statistical consequences are explicitly quantified in Section 3.4: the wide confidence interval on the primary OR (5.62–56.91) directly reflects the limited information contributed by 38 comparator observations, and multivariable models are constrained by quasi-complete separation and an events-per-variable ratio below conventional thresholds (see response to Comment 8). Confidence intervals are wide, and multivariate models may be sensitive to small data perturbations, with risk of coefficient instability and over- or underestimation of effects. This constraint is also reflected in the predictive model performance (acceptable AUC but modest positive predictive value, determined by the low event prevalence), warranting external validation on independent samples. Accordingly, the multivariable analysis is reported as exploratory: with an events-per-variable ratio of approximately 3.5, it is intended to generate hypotheses about candidate risk markers (notably older age and unemployment) rather than to provide stable, confirmatory effect estimates. The same constraint precluded fitting a strategy-adjusted multivariable model: the concentration of suboptimal events in the pharmacotherapy-alone group (9/38 vs. 5/293) would produce quasi-complete separation, making any strategy-adjusted OR unreliable. An indirect sensitivity argument based on Cornfield’s inequality is reported in Section 3.4 to partially address this gap.

Sixth, length of stay and discharge outcome may be linked through bidirectional mechanisms (severity may prolong hospitalization, while hospitalization may facilitate stabilization), introducing potential reverse causality that limits causal inference regarding the “optimal window.” This relationship may reflect a pragmatic marker of clinical complexity rather than a direct effect of duration per se.

Seventh, external validity is moderate: the study is unicentric, within a psychiatric hospital, and the cohort has a specific profile (predominantly male, concentrated in specific age groups, with a diagnostic distribution dominated by alcohol dependence). Generalization to other regions, alternative service configurations (e.g., integrated addiction/internal medicine units), or populations with different consumption patterns requires caution. In particular, alcohol dependence (F10.2) accounts for over 65% of primary diagnoses and 58% of the total cohort, while the other substance categories are more sparsely represented; consequently, our findings primarily reflect the management of severe alcohol use disorder, and their generalizability to cannabis, stimulant, or polysubstance use disorders in emergency settings will require prospective confirmation in more diagnostically balanced samples.

Eighth, the retrospective clinical documentation recorded pharmacological interventions as binary DRG-coded procedures (administered vs. not administered) without systematic patient-level data on specific drug class, dosage, duration, or route of administration. Consequently, the pharmacological component of both treatment strategies could only be characterized qualitatively (Section 2.6, Table 4) rather than through quantitative frequency distributions stratified by ICD-10 diagnosis. This precludes analysis of whether specific drug classes contributed differentially to the observed outcomes and limits comparability with studies that report detailed pharmacological protocols.

Finally, the primary outcome is assessed at discharge, without standardized longitudinal follow-up. Consequently, the persistence of abstinence, relapse rates, readmissions, mortality, and socio-professional functioning post-discharge, dimensions essential for translating acute-phase effectiveness into sustained benefit, cannot be directly quantified. The conclusions are therefore most appropriately interpreted as pragmatic evidence of short-term clinical association, with utility for triage and care organization in the emergency setting but requiring confirmation through prospective designs and medium- to long-term outcome indicators. No structured post-discharge follow-up mechanism was available for this cohort, precluding assessment of relapse, readmission, or functional outcome as a secondary endpoint in the present analysis.

### 4.4. Clinical and Organizational Implications

Given the retrospective, single-center, observational design, the following considerations should be interpreted as preliminary and hypothesis-generating rather than as definitive clinical guidance. They are offered to identify candidate directions for service organization and for prospective evaluation, not to prescribe clinical pathways. Within this framing, the findings carry potential implications for units managing acute SUD presentations, where the realistic and immediate objective is stabilization and improvement at discharge with minimization of suboptimal outcomes.

(1) Standardization of an integrated clinical pathway in emergency psychiatry. The data support the adoption of standardized protocols that combine pharmacological treatment with structured non-pharmacological components (crisis psychotherapy, motivational counseling, psychoeducation, nutritional assessment, and comprehensive paraclinical investigation) from the moment of emergency admission. These data suggest that non-pharmacological interventions may warrant consideration as core rather than optional components of care; the integrated strategy could be evaluated prospectively as a candidate default pathway, an approach that would require confirmation before implementation (*International standards for the treatment of drug use disorders*, n.d.; NICE, 2011; Louie et al., 2020; Ray et al., 2020). The relational dimension of care, including physician empathy as perceived by patients and families, constitutes an integral, measurable component of the therapeutic encounter, with direct implications for treatment engagement and adherence in acute psychiatric settings (Avram et al., 2025).

(2) Hospitalization duration as a clinical planning parameter. The identification of a 4–14-day window associated with optimal outcomes provides a pragmatic benchmark for discharge planning. As a hypothesis for prospective testing, patients discharged before day 4 might benefit from enhanced post-discharge follow-up, while hospitalization beyond 14 days could prompt reevaluation of the diagnostic formulation, investigation for occult comorbidities, and multidisciplinary case conference (Soyka et al., 2017; Tiglao et al., 2021; Wolf et al., 2020).

(3) Risk stratification and stepped care for vulnerable subgroups. The exploratory predictive model tentatively identifies patients aged ≥51 years and those who are unemployed as potential higher-risk subgroups. In practice, this supports a “step-up” care model: patients meeting these criteria may benefit from more frequent monitoring, stricter discharge criteria, pharmacological dosage adjustment (including reduction in hepatotoxic agents), and early linkage to post-discharge services—including telemedicine follow-up where feasible (Harris et al., 2019; Rehm & Shield, 2019b).

(4) Health policy framework: integration, continuity, and quality indicators. From a health policy perspective, the findings support the development of integrated emergency services (psychiatry–internal medicine–social work) with standardized triage protocols and explicit mechanisms for post-discharge continuity (“warm handoff,” rapid outpatient scheduling, functional linkage with community services). At the same time, the highly asymmetric distribution of the outcome implies that quality indicators should not be limited to “improvement at discharge” but should also encompass: rates of suboptimal outcomes, adverse events, readmissions, adherence, and medium-term functional results, representing a transition from exclusively acute-hospital indicators to care trajectory indicators (*International standards for the treatment of drug use disorders*, n.d.; NICE, 2011). Collectively, these implications are best regarded as hypotheses generated by real-world data; their translation into clinical or organizational practice should await confirmation in prospective, multicenter studies with standardized outcome assessment.

### 4.5. Future Research Directions

The present data outline a robust pragmatic signal in favor of the integrated strategy but also raise questions that must be systematically addressed to consolidate internal validity, generalizability, and operational utility.

(1) Confirmation through prospective designs and more rigorous confounding control. A priority is replication in multicenter cohorts and/or through prospective designs capable of reducing confounding by indication and residual confounding, including through analytical strategies such as propensity score matching and dedicated sensitivity analyses. The “optimal window” should be evaluated using models that more clearly separate the effect of duration from initial severity, given the potential for reverse causality between severity and length of stay.

(2) Extension of outcomes beyond discharge. Because the primary endpoint is assessed at discharge, future studies should incorporate trajectory outcomes: persistence of abstinence, relapses, readmissions, mortality, socio-professional functioning, and quality of life. A minimum follow-up period of 6–12 months post-discharge would enable a more comprehensive assessment of whether the short-term benefit associated with integrated management translates into sustained clinical and functional improvement. Establishing such a follow-up mechanism is identified as the single highest-priority extension of the present work.

(3) Granular analysis of integrated strategy components. The current operationalization treats the integrated strategy as a composite intervention. Dismantling studies or component-level analyses (e.g., psychoeducation alone vs. combined psychotherapy + paraclinical monitoring) would clarify which elements drive the observed benefit and inform resource-efficient protocol design. Future protocols could additionally incorporate modifiable nutritional biomarkers—such as vitamin D, B12, and homocysteine—given their documented association with neurodevelopmental and psychiatric vulnerability, and their potential utility as low-cost components of integrated clinical assessment (Avram et al., 2025).

(4) Substance-stratified replication. Given the pronounced dominance of alcohol dependence in our cohort, future studies should disaggregate analyses by substance category (alcohol, cannabis, stimulants, polysubstance) to test the replicability and magnitude of the integrated-strategy effect within each SUD type. Such stratified replication would clarify whether our findings reflect an alcohol-specific effect or a more general principle of emergency SUD management, informing both the targeted clinical application and the mechanistic interpretation of any confirmed associations.

(5) Sociodemographic risk scoring for emergency triage. The independent predictors identified (age ≥ 51, unemployment) could form the basis of a pragmatic sociodemographic risk score for implementation at emergency triage, enabling targeted resource allocation. Validation in independent samples and assessment of incremental predictive value over clinical variables alone are warranted.

(6) Multicenter extension across diverse healthcare systems. External validation in other Romanian centers and in healthcare systems with different organizational configurations (e.g., integrated addiction–internal medicine units, Western European emergency departments) would establish the generalizability of these findings and identify context-dependent moderators of treatment effectiveness.

## 5. Conclusions

This retrospective observational study of 331 emergency psychiatric admissions for substance use disorders (ICD-10 F10–F19) over five years (2020–2024) indicates that an integrated therapeutic strategy—pharmacotherapy combined with structured non-pharmacological interventions and paraclinical assessment—was associated with a substantially higher probability of clinical improvement at discharge compared with pharmacotherapy alone (98.3% vs. 76.3%; OR = 17.88; 95% CI: 5.62–56.91; *p* < 0.001; NNT ≈ 5). Given the observational and non-randomized design, these findings cannot establish causality; they nonetheless provide real-world evidence consistent with international guideline recommendations favouring multimodal protocols and support their consideration as a candidate clinical pathway in emergency settings managing acute substance-related presentations, pending prospective confirmation. Critically, the primary outcome is limited to clinical status at discharge; the study does not assess relapse, readmission, treatment adherence, abstinence maintenance, mortality, or any dimension of longer-term functional recovery. The observed short-term benefit may therefore not translate directly into sustained clinical or socio-professional improvement, and conclusions should be restricted to the acute-phase, in-hospital domain.

A hospitalization window of 4–14 days was associated with optimal discharge outcomes (improvement rates 99–100%), whereas both very short (≤3 days) and prolonged (≥15 days) admissions showed higher proportions of suboptimal response. Suboptimal outcomes, although rare (4.2%), were concentrated among patients aged ≥51 years (adjusted OR = 4.2; *p* = 0.001) and those who were unemployed (adjusted OR = 3.1; *p* = 0.019), providing a candidate operational basis for risk stratification at emergency triage (AUC-ROC = 0.78; sensitivity 72%; specificity 84%). Both the NNT estimate and the risk-stratification model are based exclusively on discharge-status classification; their clinical utility for longer-term outcomes—including post-discharge relapse risk, adherence to outpatient care, or readmission prevention—remains unknown and requires prospective evaluation.

These findings must be interpreted within the constraints of a single-center, non-randomized design; a small comparator group (*n* = 38 pharmacotherapy alone); an outcome restricted to clinical status at discharge without assessment of relapse, readmission, abstinence, adherence, mortality, or longer-term functioning; and a sample dominated by alcohol dependence (>65%), limiting generalizability to other substance categories. The large effect size, while statistically consistent, may reflect residual confounding by indication and should be read as an upper bound rather than a causal effect size. Future multicenter prospective studies with extended outcome assessment—relapse and readmission rates, abstinence maintenance, and quality of life at 6–12 months—and dismantling analyses of integrated strategy components are needed to establish causality, confirm generalizability, and refine evidence-based protocols for acute addiction care in emergency psychiatry.

## Figures and Tables

**Table 1:** Inclusion and exclusion criteria applied to define the analytical sample.

Criterion Type	Inclusion Criteria	Exclusion Criteria
**Diagnosis (ICD-10)**	F10–F19 code as primary or secondary diagnosis, confirmed in medical records	No ICD-10 F10–F19 code documented in the clinical file
**Admission type**	Emergency hospitalization (2020–2024)	Elective or scheduled admissions
**Age**	≥14 years at time of admission	Age < 14 years
**Data completeness**	All key variables available: intervention type, length of stay, comorbidities, discharge outcome (GOSS)	Incomplete documentation for one or more key variables; discharge outcome not recorded
**Clinical record**	Reconstructable clinical trajectory from GCOF and ER registry	Irretrievable or invalid clinical records

**Table 2:** Overview of study variables, their operationalization, and role in the analysis.

Variable	Type	Operationalization	Role in Analysis
**Age**	Continuous/Ordinal	Numeric (years); grouped: 14–18, 19–25, 26–35, 36–50, ≥51	Sociodemographic predictor
**Sex**	Binary	Male/Female	Sociodemographic predictor
**Area of residence**	Binary	Urban/Rural	Sociodemographic predictor
**Occupational status**	Categorical	Unemployed/Employed/Retired/Other	Sociodemographic predictor
**ICD-10 diagnosis**	Categorical	F10–F19 (primary vs. secondary position)	Clinical predictor
**Comorbidities**	Binary/Count	Documented psychiatric/somatic comorbidities: Yes/No	Potential confounder
**Management strategy**	Binary (exposure)	Pharmacotherapy alone vs. Integrated strategy	Primary exposure variable
**Discharge outcome (GOSS)**	Ordinal/Binary	Improved/Stationary/Worsened; recoded: Improved vs. Suboptimal	Primary outcome
**Length of stay**	Continuous/Ordinal	Days: grouped: 1–3/4–7/8–14/≥15 days	Secondary outcome/complexity marker

**Table 3:** Operational definitions of GOSS discharge categories used in the study.

Goss Category	Clinical Threshold	Clinical Description	Coding
**IMPROVED**	≥50% reduction in symptom severity vs. admission	Substantial reduction in symptom severity, psychosomatic stabilization, adequate cooperation, acceptance of therapeutic plan, including post-discharge recommendations	0 (favorable outcome)
**STATIONARY**	Variation ± 10–20%	Minimal clinical change, limited benefit, partial adherence, incomplete response to intervention	1 (suboptimal outcome)
**WORSENED**	≥30% increase in severity	Clinically relevant deterioration, relapses, symptom escalation requiring intensification of interventions, extended monitoring, or major therapeutic adjustment	1 (suboptimal outcome)

**Table 4:** Principal pharmacological classes used in the emergency management of substance use disorders, mapped to primary clinical indications and dominant ICD-10 diagnostic groups in the study cohort.

Drug Class	Examples Documented	Primary Clinical Indication	Dominant ICD-10 Groups
Benzodiazepines	Diazepam, cinolazepam, clonazepam	Withdrawal symptom control, agitation, seizure prevention	F10.2 (alcohol dependence), F13 (sedative/hypnotic disorders)
Substitution therapies	Methadone, buprenorphine	Opioid withdrawal management, stabilization	Opioid-related codes (where applicable)
Antipsychotics	As per institutional formulary	Psychotic symptoms, severe agitation, substance-induced psychosis	F12.5, F16.5, F18.5, F19.5 (substance-induced psychotic disorders)
Opioid antagonists	Naloxone	Reversal of acute opioid intoxication/overdose	Opioid intoxication (acute presentations)
Adjuvant/supportive agents	B-complex vitamins, silymarin, metoprolol	Nutritional supplementation, hepatoprotection, cardiovascular stabilization	F10.2 (alcohol dependence—thiamine/B-vitamins); cross-diagnostic (metoprolol, silymarin)

Note: This table reflects the pharmacological classes identified in the clinical documentation of the study setting. Patient-level frequency data by drug class and ICD-10 diagnosis were not available from the retrospective records; the mapping is therefore qualitative and indicative rather than quantitative.

**Table 5:** Summary of statistical methods applied in the study.

Analysis Type	Methods Applied	Purpose
**Descriptive**	Frequencies, percentages; mean ± SD; median (IQR); graphical inspection (boxplots)	Characterization of the study sample
**Inferential—categorical**	Chi-square test (χ^2^) with continuity correction; Fisher’s exact test (for small, expected frequencies)	Association between categorical/clinical/therapeutic characteristics and discharge outcomes
**Inferential—numerical**	Mann–Whitney U test; Kruskal–Wallis H test (non-parametric)	Group comparisons for continuous variables
**Effect size**	Odds Ratio (OR) with 95% Confidence Intervals	Quantification of associations
**Predictive modeling**	Binary logistic regression (suboptimal outcome as dependent variable)	Identification of independent predictors of unfavorable discharge status
**Model performance**	ROC curve analysis, AUC; sensitivity, specificity	Discriminatory performance of the logistic model
**Significance level**	α = 0.05 (two-tailed)	Applied to all inferential tests

Categorical variables were summarized using absolute frequencies and percentages; numerical variables were described through measures of central tendency and dispersion (including mean ± standard deviation and median with interquartile range, to capture potentially asymmetric distributions). Extreme values were graphically inspected (e.g., boxplots) and, when necessary, reverified against the primary clinical documents. For testing associations between demographic, clinical, and therapeutic characteristics and discharge outcomes, tests appropriate to the nature of the variables were used: chi-square (χ^2^) for comparisons between proportions (with continuity correction where applicable) and Fisher’s exact test in situations with small, expected frequencies; for comparisons of numerical variables between two or more groups, non-parametric tests were applied (Mann–Whitney U and Kruskal–Wallis H, respectively).

**Table 6:** Sociodemographic characteristics and initial clinical profile of emergency admissions for substance use disorders (2020–2024).

Domain	Category	n/N	%
**Diagnostic framing**	Primary diagnosis (F10–F19)	294/331	88.8
	Secondary diagnosis (F10–F19)	37/331	11.2
**Sex**	Male	297/331	89.7
	Female	34/331	10.3
**Age (years)**	14–18	18/331	5.4
	19–25	70/331	21.1
	26–35	89/331	26.9
	36–50	110/331	33.2
	≥51	44/331	13.3
**Area of residence**	Urban	189/331	57.1
	Rural	142/331	42.9
**Occupational status**	Unemployed	260/331	78.5
	Student	13/331	3.9
	Employed	28/331	8.5
	Disability pensioner	2/331	0.6
	Old-age pensioner	28/331	8.5
**ICD-10 profile (primary; n = 294)**	Alcohol (F10.2)	192/294	65.3
	Polysubstance (F19.*)	82/294	27.9
	Cannabinoids (F12.*)	9/294	3.1
	Sedatives/hypnotics (F13.*)	2/294	0.7
	Other stimulants/hallucinogens/solvents	9/294	3.1
**ICD-10 profile (secondary; n = 37)**	Stimulants (F15.*)	9/37	24.3
	Volatile solvents (F18.*)	9/37	24.3
	Cannabinoids (F12.*)	8/37	21.6
	Sedatives/hypnotics (F13.*)	7/37	18.9
	Hallucinogens (F16.*)	2/37	5.4
	Other (F14., F17.)	2/37	5.4
**Comorbidities**	Present	152/331	45.9
	Absent	179/331	54.1

* any subcategory within the indicated ICD-10 code (e.g., F19. includes F19.0, F19.1, F19.5, etc.).

**Table 7:** Distribution of therapeutic strategies and discharge outcome (GOSS) in the emergency cohort (N = 331).

Therapeutic Strategy	n (%)	Improved n (%)	Stationary n (%)	Worsened n (%)	Not Improved * n (%)
Pharmacotherapy alone	38 (11.5)	29 (76.3)	5 (13.2)	4 (10.5)	9 (23.7)
Integrated strategy	293 (88.5)	288 (98.3)	3 (1.0)	2 (0.7)	5 (1.7)
Total	331 (100.0)	317 (95.8)	8 (2.4)	6 (1.8)	14 (4.2)

* Not improved = Stationary + Worsened.

**Table 8:** Absolute impact of the integrated strategy on improvement at discharge (binary recoding: improved vs. not improved).

Strategy	N	Improved, n (%)	95% CI Wilson	Not Improved, n (%)	95% CI Wilson
**Integrated**	293	288 (98.3)	(96.1; 99.3)	5 (1.7)	(0.7; 3.9)
**Pharmacotherapy alone**	38	29 (76.3)	(60.8; 87.0)	9 (23.7)	(13.0; 39.2)
**Absolute difference**	—	+21.98 pp	(9.06; 38.48) †	−21.98 pp	(−38.48; −9.06)

† 95% CI for the absolute difference estimated by the Newcombe–Wilson method. pp = percentage points.

**Table 9:** Association between therapeutic strategy (integrated vs. pharmacotherapy alone) and discharge outcome: effect measures and statistical significance.

Endpoint (Operationalization)	Measure	Estimate (95% CI)	Test (2 × 2)
**Primary: Improved vs. Not improved**	OR	17.88 (5.62–56.91)	Fisher exact, *p* < 0.001
**Secondary: Not improved (Stationary + Worsened)**	RR	0.07 (0.03–0.20)	Fisher exact, *p* < 0.001
**Secondary: Worsened**	RR	0.06 (0.01–0.34)	Fisher exact, *p* = 0.002

OR = odds ratio; RR = risk ratio; 95% CI = 95% confidence interval. Fisher’s exact test was used as the principal inferential test, given low expected cell frequencies.

**Table 10:** Length of stay versus clinical outcome at discharge (primary + secondary diagnosis; N = 331).

Length of Stay	Total, n	Improved, n (%)	Stationary, n (%)	Worsened, n (%)
**1–3 days**	161	150 (93.2)	7 (4.3)	4 (2.5)
**4–7 days**	96	95 (99.0)	1 (1.0)	0 (0.0)
**8–14 days**	62	62 (100.0)	0 (0.0)	0 (0.0)
**≥15 days**	12	10 (83.3)	0 (0.0)	2 (16.7)
**Total**	331	317 (95.8)	8 (2.4)	6 (1.8)

**Table 11:** Association between comorbidities and discharge outcome (suboptimal vs. improved).

Comorbidity Status	Total (n)	Suboptimal, n/N (%; 95% CI Wilson)	Improved, n/N (%; 95% CI Wilson)
**Present**	152	9/152 (5.9%; 3.1–10.9)	143/152 (94.1%; 89.1–96.9)
**Absent**	179	5/179 (2.8%; 1.2–6.4)	174/179 (97.2%; 93.6–98.8)

Suboptimal = Stationary + Worsened. Absolute risk difference: +3.13 pp (95% CI: −1.40 to 8.33). Fisher’s exact test: *p* = 0.180.

**Table 12:** Independent sociodemographic predictors of suboptimal discharge outcome and model performance indicators (binary logistic regression).

Predictor/Indicator	Adjusted OR	95% CI	*p*-Value
**Age ≥ 51 years (vs. <51)**	4.2	1.8–9.9	0.001
**Unemployed (vs. employed)**	3.1	1.2–8.0	0.019
**Rural residence (vs. urban)**	1.4	0.6–3.3	0.42
**Male sex (vs. female)**	0.9	0.3–2.5	0.81
**Model performance**			
**AUC-ROC**	0.78	0.71–0.85	—
**Sensitivity**	72%	—	—
**Specificity**	84%	—	—
**Derived indicators**			
**Prevalence (suboptimal)**	4.2%	—	—
**PPV**	16.6%	—	—
**NPV**	98.5%	—	—
**LR+**	4.50	—	—
**LR−**	0.33	—	—

OR = odds ratio (adjusted simultaneously for all predictors); 95% CI = 95% confidence interval; AUC-ROC = area under the receiver operating characteristic curve; PPV = positive predictive value; NPV = negative predictive value; LR = likelihood ratio. Suboptimal outcome defined as stationary + worsened (prevalence: 14/331 = 4.2%).

## Data Availability

The dataset analyzed in this study consists of anonymized and ag-gregated patient data. Due to privacy and ethical restrictions, individual-level data cannot be pub-licly shared. Anonymized aggregate data may be made available from the corresponding author upon reasonable request and with permission from the institutional ethics committee.
